# Airborne Signals from a Wounded Leaf Facilitate Viral Spreading and Induce Antibacterial Resistance in Neighboring Plants

**DOI:** 10.1371/journal.ppat.1002640

**Published:** 2012-04-05

**Authors:** Yuri L. Dorokhov, Tatiana V. Komarova, Igor V. Petrunia, Olga Y. Frolova, Denis V. Pozdyshev, Yuri Y. Gleba

**Affiliations:** 1 A. N. Belozersky Institute of Physico-Chemical Biology, Moscow State University, Moscow, Russia; 2 N. I. Vavilov Institute of General Genetics, Russian Academy of Science, Moscow, Russia; 3 Nomad Bioscience GmbH, Biozentrum Halle, Halle (Saale), Germany; University of South Carolina, United States of America

## Abstract

Many plants release airborne volatile compounds in response to wounding due to pathogenic assault. These compounds serve as plant defenses and are involved in plant signaling. Here, we study the effects of pectin methylesterase (PME)-generated methanol release from wounded plants (“emitters”) on the defensive reactions of neighboring “receiver” plants. Plant leaf wounding resulted in the synthesis of PME and a spike in methanol released into the air. Gaseous methanol or vapors from wounded *PME*-transgenic plants induced resistance to the bacterial pathogen *Ralstonia solanacearum* in the leaves of non-wounded neighboring “receiver” plants. In experiments with different volatile organic compounds, gaseous methanol was the only airborne factor that could induce antibacterial resistance in neighboring plants. In an effort to understand the mechanisms by which methanol stimulates the antibacterial resistance of “receiver” plants, we constructed forward and reverse suppression subtractive hybridization cDNA libraries from *Nicotiana benthamiana* plants exposed to methanol. We identified multiple methanol-inducible genes (MIGs), most of which are involved in defense or cell-to-cell trafficking. We then isolated the most affected genes for further analysis: *β-1,3-glucanase* (*BG*), a previously unidentified gene (*MIG-21*), and *non-cell-autonomous pathway protein* (*NCAPP*). Experiments with *Tobacco mosaic virus* (TMV) and a vector encoding two tandem copies of green fluorescent protein as a tracer of cell-to-cell movement showed the increased gating capacity of plasmodesmata in the presence of *BG*, *MIG-21*, and *NCAPP*. The increased gating capacity is accompanied by enhanced TMV reproduction in the “receivers”. Overall, our data indicate that methanol emitted by a wounded plant acts as a signal that enhances antibacterial resistance and facilitates viral spread in neighboring plants.

## Author Summary

The mechanical wounding of plant leaves, which is one of the first steps in pathogen infection and herbivore attack, activates signal transduction pathways and airborne signals to fend off harmful organisms. The mechanisms by which these signals promote plant immunity remain elusive. Here, we demonstrate that plant leaf wounding results in the synthesis of a cell wall enzyme, pectin methylesterase (PME), causing the plant to release methanol into the air. Gaseous methanol or vapors from wounded *PME*-transgenic plants induced resistance to the bacterial pathogen *Ralstonia solanacearum* in the leaves of non-wounded neighboring “receiver” plants. To investigate the mechanism underlying this phenomenon, we identified the methanol inducible genes (MIGs) in *Nicotiana benthamiana*, most of which fell into the category of defense genes. We selected and isolated the following genes: *non-cell-autonomous pathway protein* (*NCAPP*), *β-1,3-glucanase* (*BG*), and the previously unidentified *MIG-21*. We demonstrated that *BG*, *MIG-21* and *NCAPP* could enhance cell-to-cell communication and *Tobacco mosaic virus* (TMV) RNA accumulation. Moreover, gaseous methanol or vapors from wounded plants increased TMV reproduction in “receivers”. Thus, methanol emitted by a wounded plant enhances antibacterial resistance as well as cell-to-cell communication that facilitate virus spreading in neighboring plants.

## Introduction

Plants are exposed to a diverse range of abiotic and biotic stresses [1]–[3]. Physical damage to a plant is a potential threat because it provides an opportunity for pathogen entry. Localized tissue damage elicits the expression of an array of antimicrobial phytochemicals [4], proteins [5], and systemic defense responses against microbial pathogens [6], [7] and herbivore attack [1], [8]–[14]. Systemic defense responses provide an attractive model for the study of cell-to-cell signal transduction pathways that operate over long distances [15], [16]. The molecular mechanisms of systemic wound signaling are not yet fully understood, but several of the non-cell autonomous signals that are released from damaged cells have been studied. In response to pathogen attack or physical damage, several plant species emit volatile organic compounds (VOCs), including ethylene [17], methyl salicylate [18], methyl jasmonate [19], [20], nitric oxide [21], [22] and *cis*-3-hexen-1-ol [23], which upregulate pathogen-related (*PR*) genes [14], [23], [24].

Pectin methylesterase (PME, EC: 3.1.1.11) [25] is a PR protein [26] and is the first barrier of defense against invading pathogens [26]–[31] and herbivores [32]–[34]. In higher plants, PME is a ubiquitous multifunctional enzymatic component of the plant cell wall (CW). The *PME* gene encodes a pro-PME precursor with an N-terminal extension of variable length [35]–[37]. The tobacco pro-PME contains a long N-terminal leader with a transmembrane domain, which is important for PME delivery into the CW [37], [38]. PME participates in CW modulation during general plant growth [39]–[42], nematode infection [43] and pollen tube growth [44]–[47].

PME interacts with the movement protein of the *Tobacco mosaic virus* (TMV) [48], [49], suggesting that PME may be involved in the cell-to-cell movement of plant viruses [50]. PME also efficiently enhances virus- and transgene-induced gene silencing (VIGS and TIGS) via the activation of siRNA and miRNA production [51], [52]. In the case of bacterial and fungal phytopathogens, PMEs act as virulence factors that are necessary for pathogen invasion and spreading through plant tissues [53]. The general structure of plant PME is very similar to that of the enzymes produced by phytopathogens [54]. Due to this structural similarity, transgenic plants overexpressing PME can be used as a model of host responses to pathogenic attack. A transgenic tobacco plant (*Nicotiana tabacum* L.) expressing a fungal PME exhibited a dwarf phenotype, modified CW metabolism [40] and a two-fold increase in leaf sap methanol levels.

The pectin demethylation directed by PME is likely to be the main source of methanol, which has long been assumed to be a metabolic waste product [55]–[57]. Methanol can accumulate in the intercellular air space at night after the stomata have closed [57]. Methanol emission peaks have been observed in the morning, when the stomata open [58]. Wounding and herbivore attack increase methanol emission levels [34], [59]–[61]. Transgenic plants with a silenced *PME* gene had a 50% reduction of PME activity in their leaves and a 70% reduction of methanol emissions compared with wild type (WT) plants. This result demonstrates that herbivore-induced methanol emissions originate from pectin demethylation by PME [33]. However, there is no direct evidence that *de novo* synthesized PME participates in methanol synthesis. In a study of VOC emissions from *Nicotiana attenuata* plants attacked by *Manduca sexta* larvae [34], [60], methanol was detected in the headspace of leaves very quickly (10 min) after leaf wounding. Therefore, it was concluded that the methanol detected was produced by PME that had been deposited in the CW before the leaf damage occurred.

To investigate the metabolism of methanol in plants, Downie et al. [62] used foliar sprays to apply methanol stimulation to *Arabidopsis thaliana* and studied the resulting changes in gene expression in leaves harvested 1, 24, and 72 h after methanol treatment using a 26,090 element oligonucleotide microarray. A concentration of 10% (v/v) methanol containing Silwet surfactant was used, to expose plants to a methanol concentration in essential excess of endogenous levels. A total of 484 (1.9%) transcripts were shown to be regulated in response to the methanol treatment. A group of genes encoding detoxification proteins, including cytochrome P450s, glucosyl transferases and members of the ABC transporter family, was the most strongly regulated group. Those authors concluded that a foliar spray of 10% methanol affects the expression of hundreds of genes, activating multiple detoxification and signaling pathways.

Here, we show that wounding results in drastic *de novo* PME synthesis. The analysis of methanol in plant emissions presents serious technical challenges. To avoid the underestimation of methanol emissions, we developed a method of methanol registration based on the high solubility of methanol in water [59]. The usage of water traps in a hermetically sealed water-drop system and a flow-through system revealed a 20-fold increase in the emission of gaseous methanol 180 min after leaf injury. To clarify the role of methanol in antibacterial resistance, we examined plant susceptibility to infection with *Ralstonia (Pseudomonas) solanacearum* [63], which causes wilt. Bacterial wilt is a devastating plant disease that affects several economically important hosts, including potatoes, tomatoes, bananas, and tobacco [64].

We showed that the methanol emitted by wounded and *PME* transgenic plants induced antibacterial resistance in non-wounded neighbor plants. We then identified more than three hundred methanol inducible genes (MIGs) that were upregulated in methanol-treated *N. benthamiana* plants. We further studied the function of three abundant MIGs: *β-1,3-glucanase* (*BG*), *non-cell-autonomous pathway protein* (*NCAPP*), and a previously unknown gene, designated *MIG-21*. Quantitative real-time PCR (qPCR) analysis of mRNA from a plant treated with gaseous methanol confirmed changes in the expression of these MIGs and revealed a specific “wave” of MIG mRNA accumulation. The wave of MIG mRNA accumulation consisted of a peak followed by attenuation. We also showed that methanol and the selected MIGs (*NCAPP*, *MIG-21*, and *β-1,3-glucanase*) induced an increase in the plasmodesmata (Pd) size exclusion limit (SEL). This was demonstrated in experiments using two tandem copies of green fluorescent protein (GFP) (2×GFP) as an indicator of Pd SEL. In addition to methanol-induced Pd gating, we also observed enhanced TMV reproduction in methanol-exposed plants and in neighbors of *PME*-transgenic and wounded plants. We hypothesize that methanol-mediated MIG upregulation and enhanced viral reproduction are unintended consequences of plant mobilization against bacterial pathogens.

## Results

### The effects of plant leaf wounding on *PME* mRNA accumulation and methanol emission

**Figure 1:**
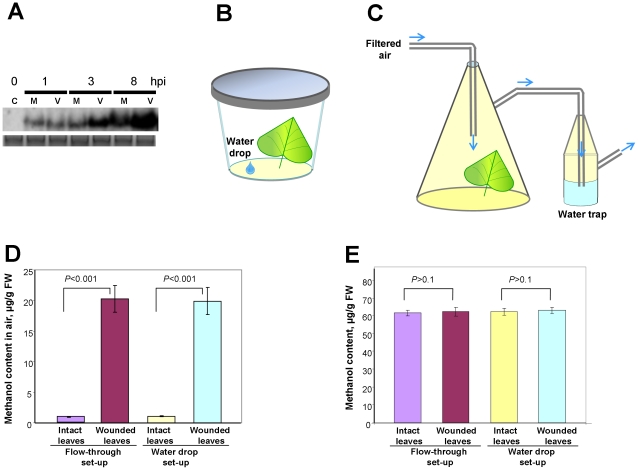
Plant leaf wounding enhances *PME* mRNA accumulation and methanol emission. (A) RNA gel blot analysis of *PME* gene expression in TMV- and mock-inoculated leaves of *N. benthamiana*. M, mock; V, TMV inoculation; C-intact control. (B, C) Schematic drawings of the experimental water-drop (B) and flow-through (C) systems used to measure the gaseous methanol emitted by intact and wounded *N. benthamiana* leaves. (D, E) Comparison of the water-drop and flow-through systems used to measure methanol in leaf headspace (D) and leaf sap (E). The data shown represent five independent experiments, with standard error bars indicated. Unpaired two-tailed Student's *t*-test *P*-values were used to assess the statistical significance of the difference in methanol production between wounded and control leaves.

Leaf wounding is often used as an experimental model of mechanical injuries sustained by a plant after wind, rain, hail, or herbivore feeding. However, serious leaf damage caused by, for example, crushing the leaf lamina with forceps [65] or puncturing leaves [34] has only a mild effect on PME gene expression. In nature, pathogen penetration of leaf tissue can occur via microdamage to the leaf cuticle, trichome or CW. Microdamage can be induced by wind-mediated leaf rubbing or insect attack. To test whether the expression of the endogenous *PME* gene is modulated by external mechanical stress, we rubbed *N. benthamiana* leaves with an abrasive water suspension of Celite. This approach is commonly used for plant virus inoculation. The 1.7-kb *PME* transcript was not detectable in intact leaves but was clearly induced after Celite rubbing at 1 hpi and was increased at 8 hpi (Figure 1A). TMV inoculation increased the accumulation of *PME* transcripts, suggesting a role for viral infection in increased *PME* mRNA levels.

We wanted to evaluate the effect of enhanced *PME* mRNA accumulation on methanol emission. We hypothesized that methanol from wounded leaves is produced by two forms of PME: pre-existing PME deposited in the CW before wounding, which allows rapid methanol release [34], [60], and PME synthesized *de novo* after wounding (Figure 1A), which likely generates methanol for an extended period (more than 8 h). Until now, the quantification of methanol emission by a plant leaf was conducted using methods based on the detection of gas-phase methanol [34], [57]–[61]. Methanol is a polar, soluble compound that is easily lost due to condensation in sampling lines and traps. Methanol mixes readily with water, a property that we exploited by using water as a trap for methanol measurement. The methanol released by wounded leaves was measured in the headspace of either a hermetically sealed jar (the water-drop system) (Figure 1B) or a glass flow chamber (Figure 1C). A drop of methanol added to the bottom of the jar will vaporize rapidly and dissolve in the water. The methanol content in the water phase may thus be used to estimate the methanol content of the leaf headspace. In the reconstruction experiment, we measured the methanol content in the water drop at different times following evaporation of various quantities of methanol that had been added to the jars. At 24°C, methanol was detected in the water drop 30 min after its addition. The water drop reached more than 80% of its saturation point after 3 h. Using calibration curves and the previously determined methanol recovery correction factors, we calculated the methanol emission of wounded leaves. Leaf wounding resulted in gaseous methanol emission, which was 20-fold higher than the methanol emission by the control intact leaf at 3 h of incubation (Figure S1). The water-drop and flow-through approaches yielded similar results for methanol emission after wounding (Figure 1D). Analysis using the unpaired two-tailed Student's *t*-test confirmed a statistically significant difference in methanol emission between the control leaves and the wounded leaves.

To determine whether methanol reabsorption might complicate our analysis, we measured the methanol content in the sap of control leaves and wounded leaves. No statistically significant methanol increase in leaf sap was detected (Figure 1E). This result indicates that essentially all methanol generated by the wounded leaves was emitted into the air.

Collectively, our data show that leaf wounding causes a rapid increase in the production of gaseous methanol.

### Methanol is required for antibacterial resistance

**Figure 2:**
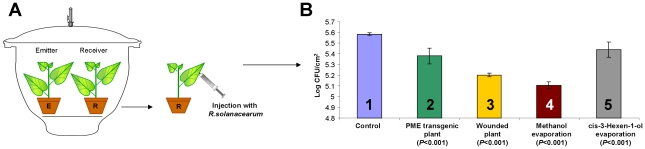
The effects of *PME* overexpression and of emitted methanol on plant resistance to *R. solanacearum*. *R. solanacearum* growth was measured in “receiver” plants incubated together with “emitter” plants, methanol or *cis*-3-hexen-1-ol in a hermetically sealed 20-l desiccator, as shown in the schematic representation (A). Wounded or *PME*-transgenic tobacco plants were used as “emitter” plants, and *N. benthamiana* plants were used as “receivers”. “Receiver” plants removed from the desiccator were tested for bacterial resistance, as shown in (B), after leaf inoculation by syringe injection with *R. solanacearum* (10^6^ cfu/ml). Log-transformed data were obtained from six independent samples. The “emitter” type and *P*-values (Student's *t*-test) are indicated under each bar.

Biologically, wound-induced *PME* gene expression and the subsequent methanol emission should lead to increased resistance to pathogens, including pathogenic bacteria [26]. To determine whether the methanol emitted by wounded plants serves as a signal for antibacterial resistance, we developed an approach (Figure 2A) in which a wounded *N. benthamiana* plant (an “emitter”) was placed in a hermetically sealed 20-l desiccator along with an intact *N. benthamiana* “receiver” plant. The “receiver” plant, which had been stored adjacent to the “emitter” plant, was removed from the desiccator, and its leaves were injected with a suspension of *R. solanacearum*, which infects a wide range of host plants. Because both whole plants were confined within the sealed container, the available CO_2_ may have been depleted. Because CO_2_ depletion could cause several types of stress, we also tested for bacterial growth in the “receiver” plant stored together with an intact plant. Figure 2B shows that, as expected, incubation with the wounded “emitter” plants led to decreased *R. solanacearum* growth in the “receiver” plants (diagram bar #3) compared with control plants (diagram bar #1). In control experiments, methanol evaporating from a piece of methanol-soaked filter paper also suppressed bacterial growth (diagram bar #4). We also tested *N. tabacum* as a “receiver” and confirmed bacterial growth suppression (data not shown).

**Figure 3:**
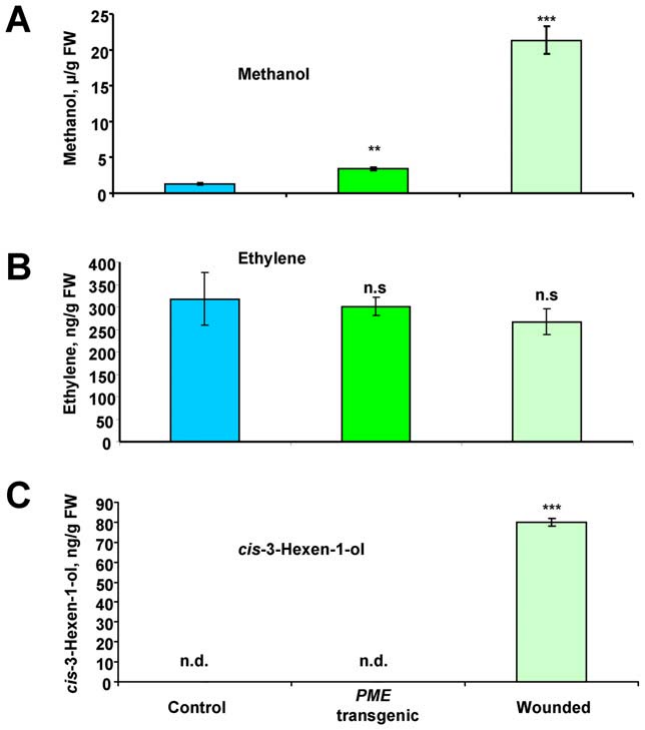
Measurement of VOCs in the headspace of *PME*-transgenic and wounded leaves. (A–C) Methanol (A), ethylene (B) and *cis*-3-hexen-1-ol (C) content detected in the headspace of a single wounded *N. tabacum* leaf or a single *PME*-transgenic tobacco leaf using the water-drop method. After a 3 h incubation, the wounded *N. tabacum* and *PME* transgenic tobacco leaves were removed, and the methanol/*cis*-3-hexen-1-ol content in the water/decane drop was measured. Ethylene was measured in the headspace air samples. Intact *N. tabacum* leaves were used as controls in this experiment. The data shown represent five independent experiments. Standard error bars are indicated. ***, *P*<0.001; **, *P*<0.01; n.s., not significantly different (Student's *t*-test); n.d., not detected.

We also examined whether green-leaf VOC (GLV) emission, which is known to be enhanced by plant wounding [34], [66]–[68], suppressed bacterial growth [69]. GLVs are lipoxygenase metabolic pathway products that include six-carbon aldehydes and alcohols. Unlike terpenoids, GLVs are rapidly, immediately and likely passively released from wounded leaves [70], [71]. Our gas chromatography (GC) analysis confirmed the presence of methanol (Figure 3A). In line with the data of von Dahl et al. [34], our GC analysis revealed that *cis*-3-hexen-1-ol is emitted in the headspace of wounded leaves (Figure 3C). We did not detect methyl salicylate or methyl jasmonate in the headspace of wounded leaves (data not shown). Ethylene emission was detected, but there was no statistically significant difference in ethylene emission between the control and wounded leaves (Figure 3B).

**Figure 4:**
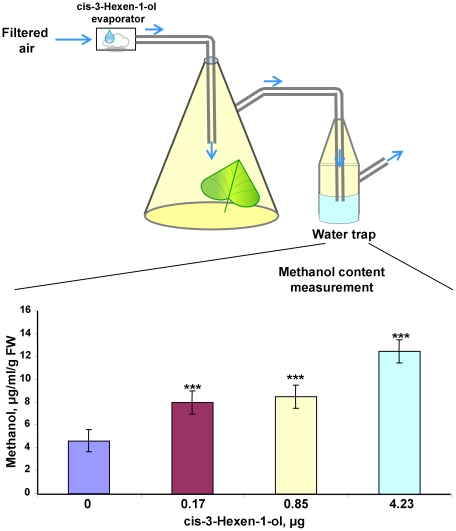
*cis*-3-hexen-1-ol vapors induce methanol emission from leaves. The upper panel depicts how *N. benthamiana* leaves were treated with a 3-h exposure to continuous airflow from an evaporator containing *cis*-3-hexen-1-ol. The bottom panel shows the methanol content in the water trap after the leaf underwent treatment with different concentrations of *cis*-3-hexen-1-ol. The data shown represent five independent experiments. Standard error bars are indicated. ***, *P*<0.001.

Thus, the suppression of *R. solanacearum* growth observed in the “receiver” plants could be caused by gaseous methanol or by GLV. Indeed, *cis*-3-hexen-1-ol evaporated in the desiccator also resulted in decreased bacterial growth in target plants (Figure 2B, diagram bar #5). However, GLVs rapidly released from wounded leaves may stimulate PME-generated methanol production, and their influence on bacterial growth may thus be indirect. To examine the role of *cis*-3-hexen-1-ol in the emission of methanol from leaves, we measured the methanol content in a water trap system in which an *N. benthamiana* leaf was exposed to continuous airflow from an evaporator containing *cis*-3-hexen-1-ol for 3 h (Figure 4, upper). The diagram (Figure 4, bottom) shows that the methanol content in the water trap increased after *cis*-3-hexen-1-ol treatment. We suggest that methanol emission induced by GLV may be responsible for the suppression of *R. solanacearum* growth in “receivers”.

To further refine the role of methanol, GLV was excluded from the gaseous mixture emitted by wounded leaves. We used the previously engineered *PME*-transgenic tobacco line, pro1, which has increased *PME* gene expression and resistance to TMV [52]. Transgenic plants produced higher levels of methanol in the leaf sap than did the control plants (Figure S2). Consistent with our expectations, the increased *PME* gene expression in the transgenic plants also resulted in a higher production of gaseous methanol, whereas *cis*-3-hexen-1-ol was not detected (Figure 3C). To determine whether the methanol emitted by *PME*-transgenic plants serves as a signal for antibacterial resistance, we employed a hermetically sealed desiccator. Incubation with the *PME*-transgenic “emitter” plants slowed the growth of *R. solanacearum* compared to the control plants (Figure 2B, diagram bar #2). Although the retardation of *R. solanacearum* growth caused by a neighboring *PME*-transgenic plant was less than that caused by a wounded plant, the reduction in growth correlated with the level of methanol emission (Figure 3A). The unpaired two-tailed Student's t-test confirmed the statistical significance of the differences in *R. solanacearum* growth retardation between the “receivers” of control and *PME* transgenic plant (Figure 2B, diagram bar #2).

**Figure 5:**
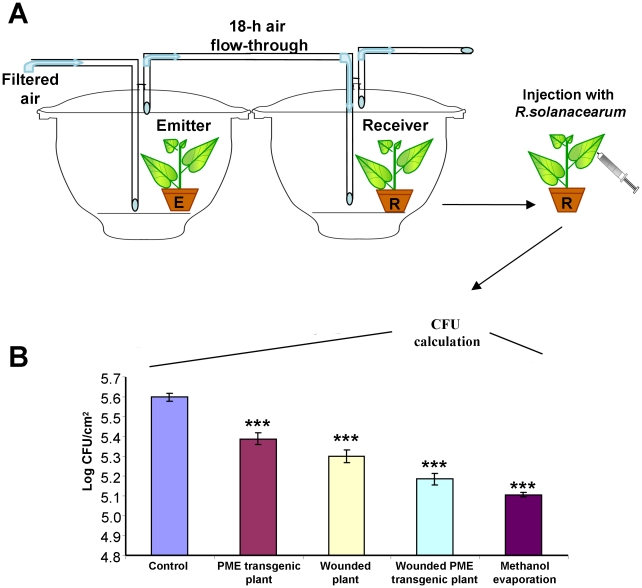
Bacterial growth is suppressed in plants maintained in a flow-through system. The measurement of *R. solanacearum* growth in “receiver” (R) plants after 3-h exposure to continuous airflow from various emitters (E): *PME* transgenic, wounded tobacco, wounded *PME-*transgenic plants or methanol evaporation in the flow-through system (A). The *N. benthamiana* “receiver” plants were inoculated by syringe injection using *R. solanacearum* (10^6^ cfu/ml) inoculum. The diagram (B) shows bacterial growth at inoculated sites on the leaves 4 days after injection with *R. solanacearum*. Log-transformed data were obtained from six independent samples. ***, *P*<0.001 (Student's *t*-test).

To further clarify the role of methanol as an airborne signal of antibacterial resistance, we again used a flow-through system that allows continuous airflow from *PME-*transgenic or wounded tobacco plants to intact target *N. benthamiana* plants (Figure 5A). Control plants were exposed to air from a desiccator containing intact *N. tabacum* plants. After exposure, the target “receiver” plants were inoculated with *R. solanacearum*. “Receiver” plants exposed to air from the desiccator with evaporated methanol, *PME*-transgenic or wounded plants acquired antibacterial resistance (Figure 5B). The evaporation from the wounded *PME*-transgenic plants had even greater effect on antibacterial activity. The unpaired two-tailed Student's *t*-test confirmed the statistical significance of the differences in decreased *R. solanacearum* growth.

Collectively, these data indicate that gaseous methanol is an airborne factor that may induce antibacterial resistance in neighboring plants.

### Identification of methanol inducible genes

In an effort to understand the mechanisms by which methanol can stimulate antibacterial resistance in “receiver” plants, we constructed forward and reverse suppression subtractive hybridization (SSH) cDNA libraries from *N. benthamiana* plants exposed to methanol.

A total of 359 differentially expressed transcripts were identified; of these, 39 appeared to be more abundant in intact leaves, and 320 appeared to be upregulated after methanol treatment (Table S1). The cloned ESTs of genes that responded to the methanol treatment were considered for sequencing. The EST sequences of the upregulated genes were deposited in the NCBI dbEST database with accession numbers. Most of the ESTs identified (Table S1) (i.e., 167) fell into the category of stress gene transcripts. We identified only one novel EST (FN432041), *methanol-inducible gene-21* (*MIG-21*) (GenBank AC GU128961), which was unrelated to all other nucleotide sequences in GenBank. *MIG-21* contains an ORF encoding a protein with a repetitive amino acid sequence (Figure S3). The methanol-specific upregulation of the SSH-identified genes was validated by a Northern blot analysis hybridized with ^32^P-labeled probes, which were prepared from randomly selected differential clones that were found by differential screening. We selected and isolated the most abundant SSH-identified genes for further analysis (Table 1) [72]–[76]. We validated the changes in gene expression observed by SSH by performing quantitative real-time PCR (qRT-PCR) to determine the mRNA levels from a plant treated with gaseous methanol. MIG mRNA accumulation depended on both the methanol concentration (Table 2) and the length of treatment (Figure S4). *PME* is not likely to be a MIG because its mRNA accumulation was not significantly altered after methanol treatment. The level of *BG* mRNA accumulation increased with time, up to 400-fold after 18 h (compared with the untreated control, Table 2). The accumulation of the *NCAPP* mRNA (GenBank AC FN432039) increased by almost 50-fold at 18 h and the level of *NCAPP* mRNA accumulation was the highest compared to *BG* and *MIG-21* after 6 h of treatment (Figure S4).

**Table 1:** The most affected SSH-identified genes selected for further analysis.

*N. benthamiana* MIGs selected for further analysis	MIG clones	MIG function	References
β-1,3-glucanase, including the vacuolar isoform and basic pathogenesis related 2 (PR-2) protein	51	Cell-to-cell movement: Pd gating regulation	72,73
Proteinase inhibitor II (PI-II)	24	Antifungal and antibacterial defense	76
Methanol-inducible gene-21 (MIG-21)	22	Cell-to-cell movement: Pd gating regulation	This article
PME inhibitor (PMEi)	7	Antifungal and antibacterial defense	75
Non-cell autonomous pathway protein (NCAPP)	3	Cell-to-cell movement	74

**Table 2:** Upregulation of *N. benthamiana* MIGs after 18 h methanol treatment.

			Relative quantity of mRNA					
Treatment	Added methanol (mg)	Methanol* content (µg/g FW)	BG	NCAPP	MIG-21	PMEi	PME	PI-II
Methanol evaporation	40	17,5**	118.70±4.01	9.89±2.67	3.60±0.80	1.92±0.63	1.03±0.33	1.32±0.42
	160	70,0**	398.53±12.93	48.84±5.19	17.51±2.60	9.71±1.31	1.54±0.47	7.11±0.68
Control	0	1.1±0.1***	1.00±0.25	1.00±0.26	1.00±0.26	1.00±0.39	1.00±0.32	1.00±0.33

*: Methanol treatment was executed by exposing two plants to methanol vapor (methanol applied to the filter paper) in a sealed desiccator. The data shown represent five independent experiments. The standard errors are indicated.

**: Methanol content in the headspace of two intact *N. benthamiana* plants (10.0±1.0 g) in a pot (width, 9.5 cm; depth, 9.5 cm) with soil (198.0±20.0 g) after an 18-h incubation.

***: Methanol content in the headspace of two intact non-treated *N. benthamiana* plants after an 18-h incubation.

**Figure 6:**
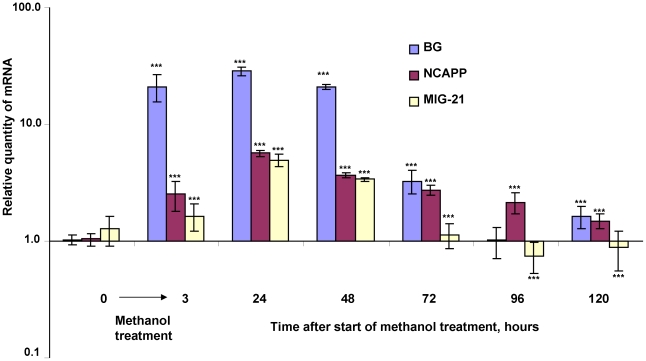
MIG mRNA accumulation peaked and then decreased after methanol treatment. The plants were subjected to methanol treatment for 3 h in a sealed desiccator with methanol (160 mg) applied to filter paper. The semi-log plot shows the measurements of the relative quantities of *BG*, *NCAPP* and *MIG-21* mRNAs, as obtained by qPCR. RNA was isolated from leaves at different times after withdrawing the plant from the methanol atmosphere. The expression level at time “0” was set to 1. The data shown represent five independent experiments, and standard error bars are indicated. ***, *P*<0.001 (Student's *t*-test).

Our model proposes that a burst of methanol from wounded leaves should elicit an extended MIG induction in neighboring leaves. We exposed *N. benthamiana* plants to methanol vapors (160 mg) applied to filter paper within a sealed 20-l desiccator for 3 h. RNA for qRT-PCR analysis was isolated from leaves at different times after the plant was withdrawn from the methanol atmosphere. Figure 6 shows the decaying wave of MIG mRNA accumulation after methanol treatment. MIGs mRNA accumulation reached a maximum at 24 h after methanol treatment and decreased slowly thereafter. Moreover, increased *BG* and *NCAPP* mRNA levels were observed as long as 5 days after methanol treatment.

**Figure 7:**
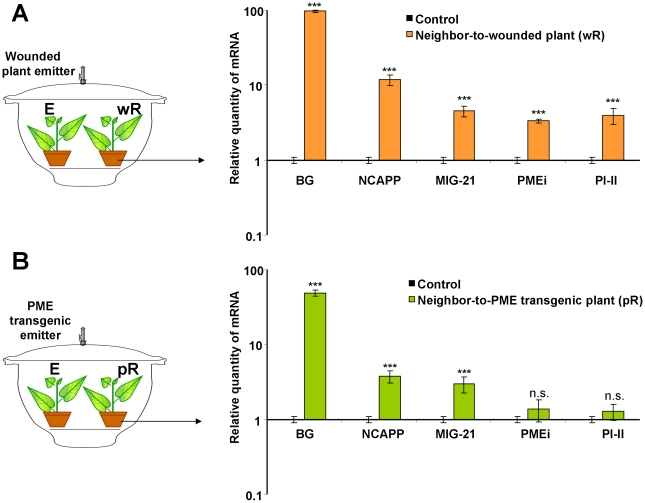
MIG transcripts accumulation is increased in the leaves of “receiver” plants after 18 h of exposure to methanol emitters. Wounded *N. tabacum* (A) or *PME*-transgenic tobacco plants (B) were used as “emitter” plants, whereas *N. benthamiana* plants were used as “receivers”, as shown in the schematic diagram (left). The semi-log plots on the right show the measurements of relative mRNA quantities for selected MIGs examined by qPCR. “Receiver” plants maintained with intact tobacco plants were used as the controls for this experiment, and the control expression level was set to 1. The data shown represent five independent experiments. Standard error bars are indicated. ***, *P*<0.001 (Student's *t*-test).

The suppression of *R. solanacearum* growth in “receiver” plants in a sealed desiccator (Figure 2B) suggests that MIGs may be involved in plant antibacterial resistance. We examined the accumulation of MIGs mRNA in *N. benthamiana* “receivers” that were kept together with wounded WT or *PME*-transgenic tobacco plants in a sealed desiccator (Figures 7 A,B). The unpaired two-tailed Student's *t*-test confirmed the statistical significance of the differences in MIG induction between the “receivers” of control and wounded or *PME*-transgenic plants.

**Figure 8:**
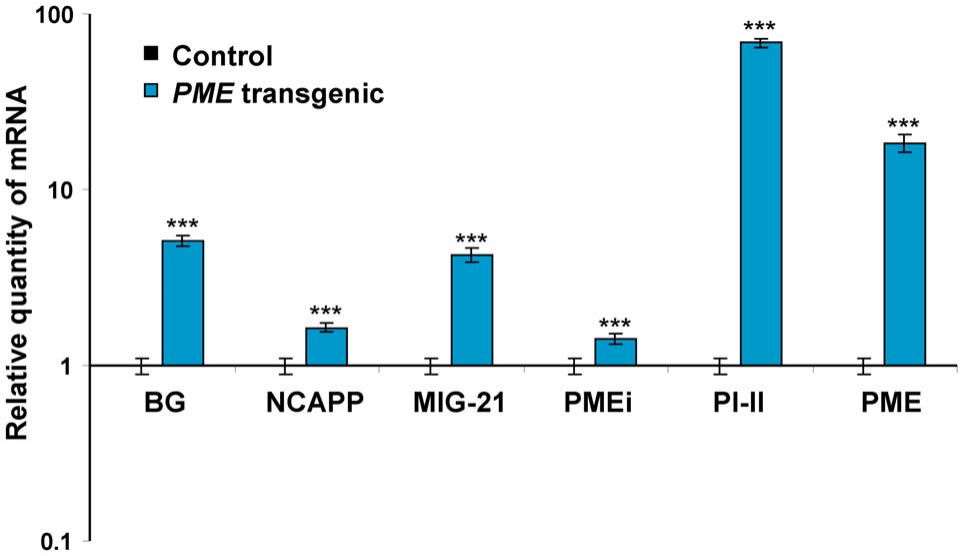
MIG expression levels were measured in the *PME*-transgenic tobacco line *pro1*. The MIG expression levels in the control plants (wild-type tobacco plants) were set to 1. The data shown represent five independent experiments. Standard error bars are indicated. ***, *P*<0.001 (Student's *t*-test).

Constant PME expression and increased methanol production in *PME*-transgenic tobacco was predicted to result in increased MIG mRNA accumulation. Indeed, RNA analysis of *PME* transgenic leaves (Figure 8) confirmed this expectation, though the general profile was different from that of methanol-treated plants. The nearly 70-fold increase observed in *PI-II* mRNA accumulation is likely to be a response to long-term PME overproduction.

It has been demonstrated previously [23] that several plant species emit VOCs, including ethylene, methyl salicylate, methyl jasmonate, and *cis*-3-hexen-1-ol, in response to pathogen attack and plant damage. In “receiver” plants, the emitted VOCs can upregulate *PR* genes, such as the basic type *PR-3* (chitinase), acidic type *PR-4* (thaumatin-like), *lipoxygenase* (LOX), *phenylalanine ammonia-lyase* (PAL), and *farnesyl pyrophosphate synthetase* (FPS). We studied gene expression in plants treated with methanol and compared those results to the gene expression of plants treated with the VOCs listed above. As shown in Figure S5, the expression of *LOX*, *PR-3*, *PR-4*, *FPS* and *PAL* genes increased slightly in methanol-treated plants. Treatment with *cis*-3-hexen-1-ol stimulated the accumulation of *FPS* mRNA, but ethylene, methyl salicylate, and methyl jasmonate treatment primarily upregulated the *PAL* and *PR-4* mRNAs accumulation.

Thus, the methanol emitted from a wounded plant most likely potentiates the antibacterial resistance of neighboring plants by increasing the MIG mRNA accumulation.

### The effect of methanol on cell-to-cell communication

Bacterial pathogens do not cross plant cell wall boundaries because they inhabit the intercellular spaces in plants. In contrast, viral pathogens require intercellular movement for local and systemic spread [16]. However, plasmodesmata (Pd) play an important role in both bacterial effector molecule spreading and host defense responses [77].

**Figure 9:**
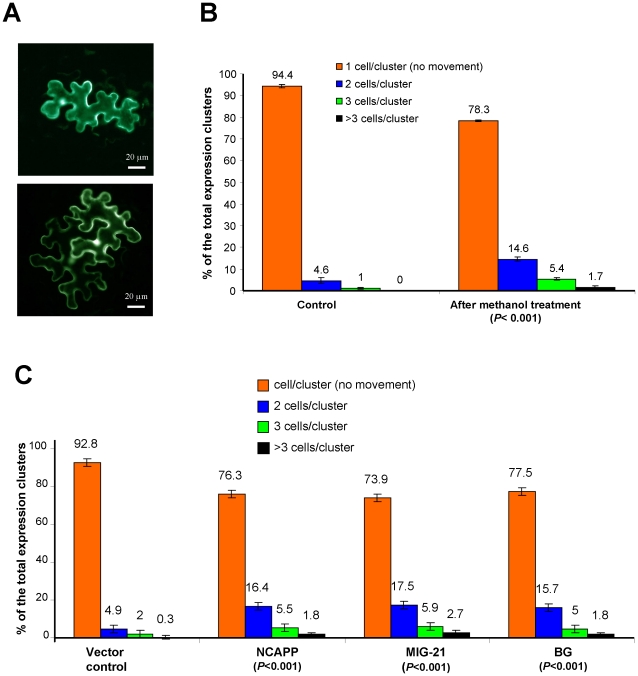
Methanol and MIGs encoding BG, NCAPP and MIG-21 facilitate cell-to-cell communication. (A) Single (upper) and multiple (bottom) epidermal cells containing 2×GFP were observed under epifluorescence microscopy after methanol treatment. (B, C) Quantification of 2×GFP movement after methanol treatment (B) and MIG agroinjection (C). No fewer than 1000 cell clusters were counted for each experiment. The data shown represent five independent experiments. Standard error bars are indicated. The unpaired two-tailed Student's *t*-test *P*-values for the statistical significance of the difference between the control and methanol-treated plants are indicated.

To evaluate cell-to-cell communication in leaves treated with methanol, a reporter macromolecule was used to test movement through Pd in different states of dilation. We chose a reporter containing two fused green fluorescent proteins (2×GFP) to query the non-targeted Pd transport of macromolecules [78]. Mature source leaves have generally been considered closed to 2×GFP (54 kDa) because their Pd size exclusion limit (SEL) does not permit proteins with a size of 47 kDa [79]. To establish a system to monitor cell-to-cell transit, we exploited an “agroinjection strategy” to deliver the 2×GFP plasmid into the cell nucleus [80], [81]. To monitor single infection sites, *N. benthamiana* plants were agroinjected with a diluted (1∶1000) bacterial suspension. Plants were then exposed to methanol vapors and examined by fluorescent light microscopy 30 h after agroinjection. Counting the number of epidermal cells surrounding the initial *Agrobacterium*-transformed cell that display fluorescence provides a quantitative measure of 2×GFP movement. When the Pd were closed, 2×GFP was detected mainly in single cells (Figure 9A, upper). However, fluorescent signals were distributed in 2- or 3-cell clusters (Figure 9A, bottom) when Pd were dilated. In the control plant, approximately 6% of the signal was distributed in 2- to 3-cell clusters (Figure 9B). These observations were consistent with the known rate of 2×GFP movement through plant tissues [78]. When methanol-treated plants were examined, more than 20% of the signal was distributed in 2 - to 3-cell clusters, indicating that the ability to support cell-to-cell movement of 2×GFP was enhanced. Specifically, whereas only 1% of the signal was found in 3 cell-clusters in the control leaves, with methanol-treatment, this value was increased up to 7%. The unpaired two-tailed Student's *t*-test confirmed the statistical significance of the differences in the cell-to-cell movement of 2×GFP between the control plant and plants treated with methanol (Figure 9B).

Collectively, these data indicate that methanol acts as a signal that facilitates the movement of 2×GFP between cells.

To examine the role of MIGs in Pd dilation (gating), we monitored the relative cell-to-cell spreading of 2×GFP within the epidermis of *N. benthamiana* leaves co-agroinjected with binary plasmids encoding *BG*, *NCAPP* or *MIG-21* directing the synthesis of the respective mRNAs, as tested by qRT-PCR (data not shown). Figure 9C shows that in the control leaves, which were co-agroinjected with an empty Bin19 vector, approximately 7% of the signal was distributed in 2- to 3-cell clusters. When leaves were co-agroinjected with *NCAPP*, *MIG-21* or *BG*, the movement of 2×GFP was enhanced: more than 23, 26 or 22% of the 2×GFP signal was detected in cell clusters. The unpaired two-tailed Student's *t*-test confirmed the statistical significance of the differences in the cell-to-cell movement of 2×GFP between the vector-only control and leaves co-agroinjected with *NCAPP*, *MIG-21* or *BG* (Figure 9C).

Collectively, these data imply that gaseous methanol may trigger leaf Pd dilation (gating) by inducing the mRNA accumulation of MIGs such as *NCAPP*, *MIG-21* and *BG*.

### Methanol facilitates TMV infection

**Figure 10:**
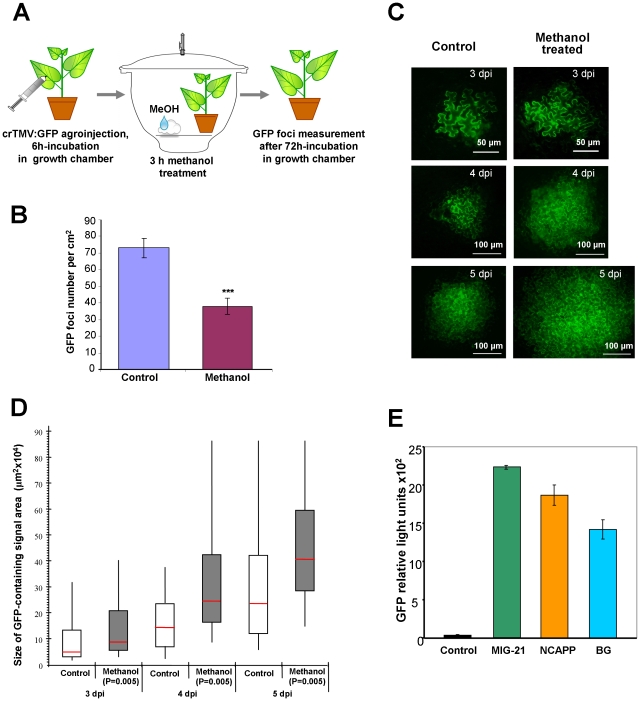
The local movement of crTMV:GFP is facilitated in methanol-treated leaves. (A) Schematic drawing of the experimental procedures used to measure the local movement of crTMV:GFP in methanol-treated leaves. (B) Quantification of crTMV:GFP foci in the leaves of control and methanol-treated plants at 3 dpi. No fewer than 1000 foci were counted. The data shown represent five independent experiments. Standard error bars are indicated. The unpaired two-tailed Student's *t*-test *P*-values for the statistical significance of the difference between the control and methanol-treated plants are indicated. ***, *P*<0.001. (C) Epifluorescent micrographs of crTMV:GFP foci in control (left panel) and methanol-treated (right panel) plants. (D) Box-and-whisker plots show the statistical distribution of the data, and horizontal red lines across the boxes represent the median size of the crTMV:GFP expression foci (µm^2^×10^4^). The ends of the boxes indicate the 1^st^ and 3^rd^ quartiles, respectively. No fewer than 1000 foci were counted. The data shown represent five independent experiments. The statistical significance of the differences between each control and methanol-treated plant was analyzed by ANOVA and calculated to be *P* = 0.005. (E) MIG co-injection enhances crTMV:GFP vector reproduction. A fluorimetric analysis of GFP accumulation was conducted using leaves grown for 5 days after co-agroinjection with crTMV:GFP and vectors encoding BG, NCAPP and MIG-21. Fluorescence measurements are presented in relative light units. The GFP fluorescence, which was observed in co-agroinjection with empty pBin19, was assigned a value of 1. Empty pBin19 served as a control. The data shown represent 5 independent experiments. Standard error bars are indicated.

Our model suggests that methanol-triggered Pd dilation should enhance viral spread within the plant. To examine this possibility, we inoculated plants with a crTMV binary vector that carries an autofluorescent tag GFP (crTMV:GFP) in the place of its *coat protein* gene [82] and treated the transfected plants with methanol, as shown in Figure 10A. Figure 10B shows the quantification of GFP foci in leaves at 3 dpi. Methanol treatment reduced the number of GFP foci per cm^2^ presumably due to the induction of antibacterial resistance, which was consistent with our data showing that methanol exposure inhibited *R. solanacearum* growth (see Figure 2B). Importantly, the stimulation of local viral movement by methanol was indicated by the appearance and spread of the GFP signal. Figure 10C shows that while viral foci became visible in all plants approximately at the same time (3 dpi) after inoculation, viral reproduction representing viral RNA replication and RNA cell-to-cell movement occurred more rapidly in the methanol-treated plants than in the control plants. Figure 10D summarizes the results of the statistical analysis of the data, with the horizontal red lines across the boxes representing the median size of the GFP expression foci (µm^2^×10^4^). ANOVA confirmed the statistical significance of the differences in focus size between the control and methanol-treated leaves (*P* = 0.005).

Because BG, NCAPP and MIG-21 can enhance cell-to-cell movement, they may also increase viral RNA movement and/or replication. Therefore, BG, NCAPP and MIG-21 may increase TMV-directed GFP accumulation due to viral reproduction. We tested this hypothesis using crTMV:GFP and binary vectors encoding BG, NCAPP and MIG-21 through co-agroinjection of *N. benthamiana* leaves. At five days after co-agroinjection with vectors encoding BG, NCAPP and MIG-21, the GFP accumulation in whole leaves increased by 13–23 fold (Figure 10E). These results suggest that BG, NCAPP and MIG-21 enhance viral reproduction. A change in the accumulation of GFP expressed from the viral vector can be caused by a change in viral RNA movement and/or a change in viral replication.

Under natural conditions, viral RNA directly enters the cytoplasm of a negligible number of cells following leaf wounding. *Agrobacterium*-delivered plant viral vectors exploit the host RNA polymerase II–mediated nuclear export system, which includes 5′-end capping, splicing and 3′-end formation [83]. To test whether methanol or vapors from wounded plants can enhance viral reproduction in TMV-inoculated leaves, we used an experimental design that mimicked the natural condition of viral entry, excluding *Agrobacterial* participation.

**Figure 11:**
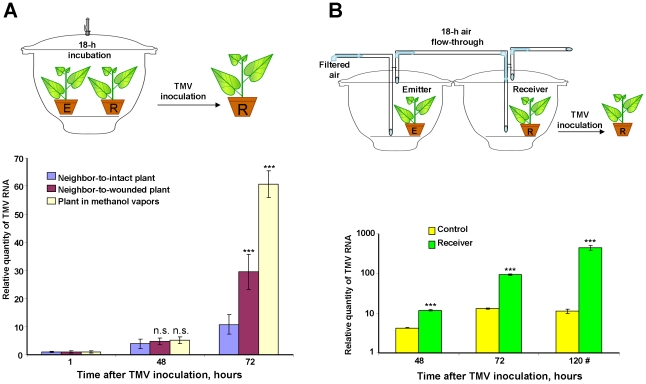
Effects of emitted methanol on plant sensitivity to TMV. (A) TMV RNA accumulation in a “receiver” (R) plant maintained together with an “emitter” (E) plant in a hermetically sealed 20-l desiccator was measured as depicted in the schematic representation (upper panel). Wounded *N. benthamiana* plants were used as “emitters”, and intact *N. benthamiana* plants were used as “receivers”. RNA samples isolated 1, 48 and 72 h after leaf inoculation were analyzed by qRT-PCR using TMV *MP* gene-specific primers. Methanol (160 mg) applied to filter paper was used for the positive control. The data shown represent five independent experiments. *** *P*<0.001, n.s. = not significant (Student's *t*-test). (B) Measurement of TMV RNA accumulation in “receiver” (R) plants after exposure to continuous airflow from emitters (E), i.e., wounded *N. benthamiana* plants, in the flow-through system (upper panel). The semi-log plot (bottom panel) shows the relative quantities of the TMV RNA accumulated in inoculated (48 and 72 h) and systemic upper leaves (120 #). The relative quantities of TMV RNA were determined by qRT-PCR using TMV *MP* gene-specific primers. The data represent five independent experiments. Standard error bars are indicated. *** *P*<0.001 (Student's *t*-test).

In contrast to controls, plants incubated with wounded *N. benthamiana* in a hermetically sealed desiccator exhibited increased sensitivity to TMV, as reflected by TMV RNA accumulation (Figure 11A). The same effect occurred when methanol was evaporated in the desiccator. The unpaired two-tailed Student's *t*-test confirmed the statistical significance of the differences in TMV RNA accumulation between the “receivers” of intact plants, wounded plants or methanol.

The use of the flow-through system to provide continuous airflow from wounded *N. benthamiana* plants to intact target *N. benthamiana* plants (Figure 11B, upper panel) confirmed the results of experiments with the hermetically sealed desiccator. “Receiver” plants exposed to air from the desiccator containing wounded plants acquired increased sensitivity to TMV in comparison to control plants (Figure 11B, bottom).

The statistical significance of the differences in TMV RNA accumulation in the inoculated (48 and 72 h after TMV inoculation) or systemically infected leaves (120 h after TMV inoculation) between the “receivers” of intact or wounded plants were confirmed by the unpaired Student's *t*-test.

These data indicate a role for methanol in triggering MIG expression, which leads to enhanced viral spread and/or reproduction.

## Discussion

The amazing capacity of plants to recognize pathogens through strategies that involve both conserved and variable pathogen elicitors has been previously reported [5], [84], [85]. However, the molecular mechanism by which plants protect themselves against bacterial pathogens remains obscure. This is mainly due to a lack of knowledge about the long-distance signals that trigger systemic reactions in plants. One recent study suggested that a long-range factor, GLV, may increase resistance to the bacterial pathogen *Pseudomonas syringae* [68]. Here, we characterized another VOC, methanol, which induces a protective reaction against *R. solanacearum*.

Methanol is a natural plant product that accumulates in the leaf tissue and is emitted when the stomata open in the morning [57], [58]. Our data reveal that leaf wounding stimulates additional methanol emission. Five aspects of wound-stimulated methanol production are especially interesting. First, there is a direct correlation between *de novo* PME synthesis and methanol emission (Figure 1A,D). We observed a 20-fold increase in the emission of gaseous methanol at 3 h after leaf damage in comparison to the methanol emission by intact control leaves (Figure 1D). Second, methanol generated by *de novo* synthesized PME is released into the air but does not accumulate in leaf tissue or sap (Figure 1E). Third, gaseous methanol upregulates methanol-inducible genes (MIGs) in the leaves of neighboring plants (Figures 6,7). Fourth, methanol induces antibacterial resistance (Figures 2,5). Fifth, although virus entry *per se* induces PME mRNA accumulation (Figure 1A), gaseous methanol drastically increases the TMV sensitivity of non-wounded leaves (Figure 11).

**Figure 12:**
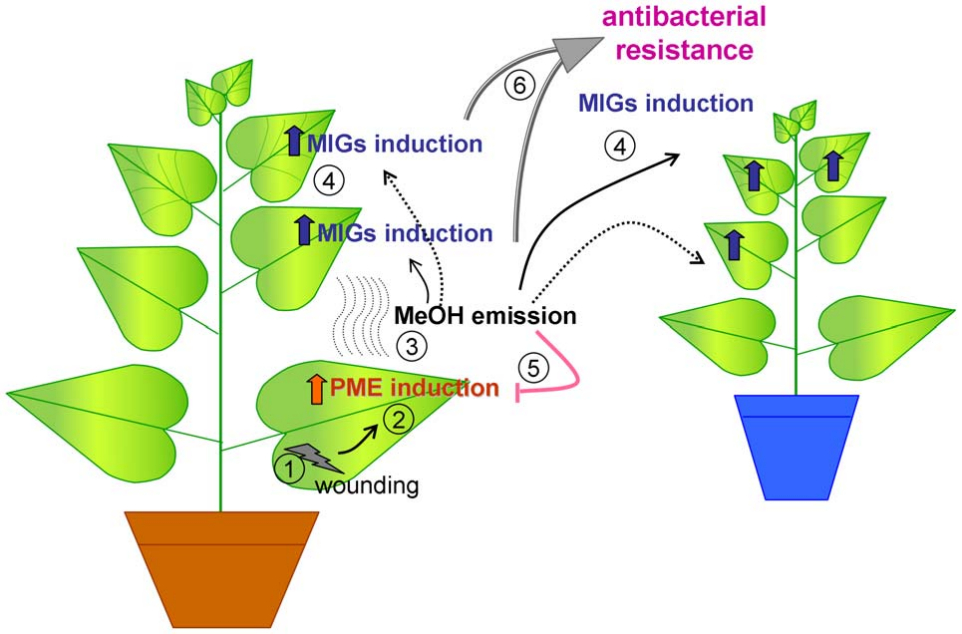
The putative model for the effect of methanol release after plant wounding. Microwounding of a plant leaf (1) leads to an increase in *PME* expression levels (2), followed by methanol emission in the wounded tissue (3). Plant-emitted methanol induces MIG expression in the other leaves of the wounded plant and the leaves of neighboring plants (4). MIG induction leads to transient antibacterial resistance in the wounded plant and neighboring plants (6). Feedback between methanol emission and PME induction is indicated (5).

We suggest the following model to explain the mechanism of the observed phenomenon (Figure 12). Microdamage (Figure 12, step 1) to the leaf caused by wind-induced leaf rubbing, human handling or insect attack, results in the upregulation of the *PME* gene (Figure 12, step 2). Upregulation of the *PME* gene leads to at least three events. First, PME triggers defense reactions that provide resistance against bacteria and viruses; i.e., wound mediated *PME* mRNA accumulation may promote the defense reactions described earlier [52]. It is worth to emphasize that a model for a mechanical damage – transgenic tobacco overexpressing *PME* – is resistant to *R. solanacearum* (see Figure S6 and Table S2). Second, PME enzymatic activity increased 2.5-fold in *N. benthamiana* leaves at 3 h after wounding (139±9.2 vs. 360±0.068 nkat/mg). Third, PME catalyzes the production of gaseous methanol (Figure 12, step 3), which induces the MIG mRNA accumulation (Figure 12, step 4). Gaseous methanol may provide a feedback loop and suppress *PME* transcription (Figure 12, step 5) such that the leaf returns to its pre-wounding methanol production state. PMEi is likely to take part in this process by suppressing PME enzymatic activity [75]. MIGs are responsible for TMV spreading/reproduction and resistance to *R. solanacearum* (Figure 12, step 6).

It was previously shown that transgenic tobacco with elevated *PME* synthesis is resistant to TMV [52]. This strain exhibits increased methanol emission levels and MIG expression but is not susceptible to TMV. We can consider the effects in PME-transgenic plants to be a consequence of long-term (even “lifelong”) MIG induction, which clearly differs from the effects of short-term methanol treatment. These cases are thus examples of ‘chronic’ and ‘acute’ situations, respectively. The patterns of MIG expression in these two cases are similar (i.e., activated) but still very different (compare Figures 6 and 8). Methanol treatment elicits a MIG “wave” that eventually fades, while MIG expression in *PME*-transgenic plants is always slightly elevated, which might lead to some secondary effects. Moreover, the expression of PME is much higher in *PME*-transgenic plants than in methanol-treated plants. We believe that this increased PME expression, which is absent in methanol-treated plants, makes the PME-transgenic plant resistant to TMV.

Methanol is not a plant poison. Treatment of plants with high-concentration methanol solutions (5–50%) revealed that foliar sprays of aqueous methanol, even at a concentration of 50%, led to increased growth and development in C_3_ crop plants in arid environments [86]. This is likely to be the result of more effective utilization of light energy during photosynthesis [87].

Previously, foliar sprays of a 10% methanol solution were used to identify methanol-sensitive genes in *Arabidopsis thaliana* [62]. Methanol affected the expression of hundreds of genes, and multiple detoxification and signaling pathways were activated. We used gaseous methanol at physiological concentrations, which were likely 10,000 times lower than those used by Downie *et al.* [62]. This difference in methanol concentration may explain why we observed the upregulation of only a few previously identified genes (see Table S1). Most of the MIGs identified here (167 ESTs) were classified as stress response genes. The majority of these (117 clones) represented 6 of the most up-regulated SSH-identified genes: *BG*, *PI-II*, *MIG-21*, *PMEi*, *elicitor inducible protein* and *1-aminocyclopropane-1-carboxylic acid oxidase*, the latter of which is involved in ethylene biosynthesis [88]. The *NCAPP* transcript was represented by only three clones (Table S1); however, qRT-PCR verification (Table 2) showed that the *NCAPP* gene was highly inducible, the second most inducible after *BG*. The SSH approach did not identify the *LOX*, *PR-3*, *PR-4*, *FPS* or *PAL* genes, which are induced by other VOCs (Figure S5).

Pathogen attack and plant damage accompanied by the emission of VOCs, including ethylene [17], methyl salicylate [18], methyl jasmonate [19], [20], nitric oxide [21], [22] and *cis*-3-hexen-1-ol [23], leads to the upregulation of different *PR* genes [14], [23], [24]. In addition to methanol, we detected the emission of ethylene and GLV. Ethylene is a simple gaseous hormone that integrates external signals with internal processes. Wound-induced ethylene production has been studied thoroughly [89]. The two-step ethylene biosynthesis, i.e., the conversion of S-adenosyl-L-methionine to 1-aminocyclopropane-1-carboxylic acid (ACC) and its subsequent oxidation to ethylene, is regulated by ACC synthase (ACS) and ACC oxidase (ACO), respectively. ACS and ACO are encoded by members of multi-gene families [90]–[92]. Ethylene production is regulated by different isoforms of ACO and ACS in response to different stresses [93]. For example, the accumulation of the transcripts of 3 out of 4 members of the ACO gene family has been examined in tomato, and only ACO1 was wound-responsive [90]. Our SSH approach revealed 6 ACO clones in leaves treated with methanol. We also showed that leaf wounding or PME overexpression (Figure 3) did not increase ethylene emission as a secondary response to methanol. This contradiction might be explained by previous data indicating that ACC synthase, but not ACO, is rate-limiting in ethylene biosynthesis [94]. We have not detected ACC synthase gene upregulation (Table S1). Moreover, it has been shown that increased ACO activity does not always immediately lead to parallel changes in ethylene production [95], e.g., in stress response (methylviologen, oxidative stress inductor, or methyl jasmonate). We hypothesize that methanol might be a similar stimulus, affecting ACO but not ethylene synthesis when applied at physiological concentrations. On the other hand, ethylene biosynthesis is regulated by different isoforms of ACO in response to particular stress cues [93]. Finally, the antibacterial effects of methanol were demonstrated not only in sealed desiccators but also in a flow-through system (Figure 5) in which methanol was blown out. Therefore, the effects of virtual ethylene were excluded or at least significantly diminished.

We also detected *cis*-3-hexen-1-ol as a representative of GLV emission after plant wounding (Figure 3). The suppression of *R. solanacearum* growth observed in the “receiver” plants could be caused by gaseous methanol and GLV. This was confirmed in experiments in which *cis*-3-hexen-1-ol evaporated in the desiccator resulted in decreased bacterial growth in target plants (Figure 2B, diagram bar #5). In an attempt to elucidate the mechanism underlying this phenomenon, we discovered that GLVs rapidly released from wounded leaves stimulate PME mRNA accumulation and therefore PME-generated methanol emission. In our experiments with detached *N. benthamiana* leaves incubated for 3 h in a 300-ml sealed container with *cis*-3-hexen-1-ol (0.36 µg), the level of PME mRNA increased by more than two times (2.41±0.37) in comparison to water control (1.00±0.25). Taking into account the connection between *cis*-3-hexen-1-ol, PME and methanol emission, we believe that the effect of *cis*-3-hexen-1-ol on bacterial growth is indirect.

Antibacterial resistance accompanied by MIG upregulation is likely to be related to *PI-II* gene transcription induction. Type I proteinase inhibitors are powerful inhibitors of serine endopeptidases in animals and microorganisms [96]. The *PI-II* gene is not expressed in the leaves of healthy plants, but it is induced in leaves that have been subjected to different types of stress, including wounding and bacterial infection [76]. *R. solanacearum* encodes several secreted proteases [97], [98], including a type III effector, *PopP2*, which mimics a plant transcriptional activator and manipulates the plant transcriptome [99], [100]. *PME*-transgenic tobacco with high levels of *PI-II* expression (Figure 8) demonstrated high resistance to *R. solanacearum* (Figure S6 and Table S2). This finding supports the role of PI-II in the suppression of bacterial proteases.

To determine whether *BG*, *MIG-21* and *NCAPP* could enhance cell-to-cell communication, we used *Agrobacterium* to mediate the delivery of GFP- and MIG-expressing vectors. Although methanol treatment induced resistance against bacteria (Figure 10B) and therefore decreased the number of detected crTMV:GFP foci, we found that these foci increased in size (Figure 10 C,D). Methanol changes Pd SEL and upregulates MIGs (*BG* and *NCAPP*); therefore, it is likely to promote cell-to-cell trafficking and TMV reproduction. The participation of MIG-21 in cell-to-cell trafficking is unconfirmed, but the role of BG and NCAPP in Pd dilation has been described previously [74], [101], [102]. However, there is no data explaining the correlation between methanol-mediated *BG*, *NCAPP*, *MIG-21* upregulation and antibacterial resistance. A recently revealed link between nuclear transport and cell-to-cell movement [103] suggests that there may be competition between methanol-mediated cell-to-cell transport and *R. solanacearum* type III effector nuclear traffic. We cannot exclude the possibility that airborne signals from wounded leaves may also facilitate TMV spreading/reproduction in neighbors as an unintended consequence of the acquired antibacterial resistance of the plants. Interestingly, it has been suggested that the conditions generated by agriculture during the Holocene period may have promoted viral spreading in plants [104].

Further research is required to elucidate the mechanisms of the reactions triggered by methanol in plants. How methanol is regulated during wound stress conditions remains unclear, as do the identities of possible factors involved in this process. The involvement of MIGs in viral spreading has been clearly demonstrated. However, the underlying cellular mechanisms controlling the targeting of BG, NCAPP and MIG-21 to the Pd is still unknown. Finally, the factors that coordinate the spatiotemporal correlation of MIGs with bacterial resistance and viral cell-to-cell spreading and reproduction have yet to be determined.

## Materials and Methods

### Plant growth conditions

*N. benthamiana* and *N. tabacum* plants were grown in soil in a controlled environment chamber in a 16 h/8 h day/night cycle.

### Plasmid and vectors

Full-length *BG* (*β-1,3-glucanase*), *MIG-21* and *NCAPP* cDNAs were obtained by PCR using the primer pairs 5′GAGCTCATGTCTACCTCACATAAACATAATAC3′/5′AAGCAGTGGTAACAACGCAGAGTACtttttttttttttttttttttttttttttt3′, 5′GAGCTCATGGCATCACTTCAGTGCC3′/5′CTGCAGTCAGCAGCTCCCTCTATTC3′ and 5′GAGCTCATGTCTTCAAAGATTGGTCTG3′/5′CTGCAGCTATTTCTTGATAGAAAACGTG3′, respectively, with total *N. benthamiana* cDNA as the template.

The viral vector crTMV:GFP (pICH4351) has been described previously [105]. To synthesize the 35S-based binary vectors pBG, pMIG-21 and pNCAPP, PCR-amplified cDNA was inserted into the XbaI, EcoRI (pBG) or SacI, PstI (pMIG-21 and pNCAPP) sites of pBin19.

### Single-leaf VOC measurements

#### Methanol analysis

The methanol emitted by wounded leaves was measured in the headspace of either hermetically sealed jars (water-drop set-up) or glass flow chambers using a water sample as a trap. The water-drop set-up was achieved using hermetically sealed plastic jars (150 ml) with a drop of water (300 µl). *N. benthamiana* leaves (0.5–0.7 g) were rubbed with Celite and loaded into the jar. After a 30, 60 or 180-min incubation, the leaves were removed, and the methanol contents in the water drop and leaf sap were examined.

The flow-through set-up included a glass flow chamber (300 ml) supplied with filtered air at a rate of 0.15 l/min attached to two tubes with a 1-ml water trap placed on ice. After a 180-min incubation at 24°C, the leaves were removed, and the methanol contents in the water traps and leaf sap were examined.

To analyze the methanol content of plant sap, 30 mg of leaf tissue was ground in the presence of Celite in microtubes and centrifuged at 16,000× g for 10 min. An equal volume of 10% trichloroacetic acid was added to each supernatant aliquot. After a 20-min incubation on ice, samples were centrifuged at 16,000× g for 15 min, and 2 µl of the supernatant was used for GC analysis.

Methanol content was determined by GC using the calibration curves obtained for water-drop jars and glass flow chambers by repeated injections of 0.25, 0.35, 0.5, 1, 10, 50 and 100 µl of methanol into jars and glass flow chambers containing leaf material. Methanol emissions are expressed as µg methanol per 1 g fresh weight of leaf.

#### Analysis of *cis*-3-hexenol

*cis*-3-hexen-1-ol was measured in hermetically sealed 150-ml jars with a 300-µl drop of decane. After a 3 h-incubation at 24°C, the jar with leaves was opened, and the *cis*-3-hexen-1-ol content in the decane drop was measured by GC using the calibration curve obtained by repeated injections of 0.05, 0.5, 5.0, and 50 µg of *cis*-3-hexen-1-ol (Sigma-Aldrich, USA) into jars containing leaf material. *cis*-3-hexen-1-ol emissions are expressed as ng *cis*-3-hexen-1-ol per 1 g fresh weight of leaf.

#### Ethylene analysis

Ethylene was measured in the headspace of a single leaf in 150-ml jars. Ethylene for the calibration curve was obtained by the reaction of 10 M KOH and ethephon (Sigma-Aldrich,USA).

### GC analysis

The methanol, *cis*-3-hexen-1-ol and ethylene contents were determined by GC on a capillary FFAP column (50 m×0.32 mm; Varian Inc., Lake Forest, CA, USA) in a Kristall 2000 gas chromatograph (Eridan, Russia). The methanol and *cis*-3-hexen-1-ol in water/decane samples were measured under the following operating conditions: carrier gas – nitrogen, nitrogen flow – 30 ml/min; air flow – 400 ml/min; hydrogen flow – 40 ml/min; injected volume – 1 µl; injector temperature – 160°C; column temperature – 75°C, increased to 150°C at a rate of 15°C/min; retention time – 6.5 min (methanol) or 17 min (*cis*-3-hexen-1-ol); and flame ionization detector temperature – 240°C. Ethylene content in air samples was analyzed under the following operating conditions: carrier gas – nitrogen; nitrogen flow – 30 ml/min; air flow - 400 ml/min; hydrogen flow – 40 ml/min; injected volume – 1 ml of vapor phase; injector temperature – 130°C; column temperature – 45°C; retention time – 4.5 min; and flame ionization detector temperature – 240°C.

### Whole plant methanol treatment and measurement

Methanol treatment was executed by exposing plants to methanol vapors on filter paper in a sealed desiccator. The effects of plant VOCs were measured in either a single hermetically sealed 20-l desiccator or a flow-through set-up involving two attached 20-l desiccators (the first for the “emitter” plants and the second for the “receiver” plants) supplied with filtered air at a rate of 0.15 l/min. Intact and wounded *N. tabacum* or *PME*-transgenic tobacco plants were used as “emitter” plants, whereas *N. benthamiana* plants were used as “receivers”. Pots (width, 9.5 cm; depth, 9.5 cm) containing plants (10.0±1.0 g) and soil (198.0±20.0 g) were placed into desiccators and maintained for 3 h or 18 h at a constant temperature of 24°C with a 16 h/8 h light/dark photoperiod. Then, “receiver” plants were withdrawn from the desiccator and tested for MIG RNA accumulation and bacterial and TMV resistance. In experiments assessing the decay of MIG mRNA accumulation, the plants withdrawn from the desiccator after methanol treatment were kept at 24°C with a 16 h/8 h light/dark photoperiod for leaf RNA isolation.

### Plant infection with *R. solanacearum*

The tobacco strain *R. solanacearum* was grown under routine conditions on yeast–peptone–glucose (YPG) agar containing the following (per liter): 5 g yeast extract, 10 g peptone, 5 g glucose and 15 g agar. The incubation temperature was 28°C. Overnight cultures of *R. solanacearum* at the indicated concentrations in 10 mM MES (pH 5.5) buffer supplemented with 10 mM MgCl_2_ were injected into fully developed leaves by syringe. At four days post inoculation (dpi), bacterial growth was measured by macerating five leaf discs of 1 cm^2^ from the inoculated tissue of each sample in 10 mM MgCl_2_, plating the serial dilutions on nutrient agar plates, and counting the colony-forming units (cfu).

### Agroinjection experiments

*Agrobacterium tumefaciens* strain GV3101 was transformed with individual binary constructs and grown at 28°C in LB medium supplemented with 50 mg/l rifampicin, 25 mg/l gentamycin and 50 mg/l carbenicillin/kanamycin. *Agrobacterium* cells from an overnight culture (5 ml) were collected by centrifugation (10 min, 4,500× g), resuspended in 10 mM MES (pH 5.5) buffer supplemented with 10 mM MgSO_4_ and adjusted to a final OD_600_ of 0.2 for TMV-directed GFP accumulation or 0.001 for cell-to-cell movement assays. Agroinjection was performed on almost fully expanded *N. benthamiana* leaves that were still attached to the intact plant. A bacterial suspension was infiltrated into the leaf tissue using a 2 ml syringe, after which the plants were grown under greenhouse conditions at 24°C with a 16 h/8 h light/dark photoperiod. In the cell-to-cell-movement assay, *N. benthamiana* plant leaves were agroinjected with 2×GFP and were stored for 6 h in a plant growth chamber at 24°C with light; these plants were then loaded into the desiccator. Then, methanol (160 mg) was added, and the desiccator was sealed. After a 3-h exposure to methanol vapors, the plants were withdrawn, and fluorescent cells were counted after 21 h of storage in a growth chamber. In the viral focal growth experiments assessing TMV cell-to-cell spreading, *N. benthamiana* plant leaves were agroinjected with crTMV:GFP, stored for 6 h in a plant growth chamber, and then loaded in the desiccator. Subsequently, methanol (160 mg) was added, and the desiccator was sealed. After a 3-h exposure to the methanol vapors, the plants were withdrawn. Fluorescent cells were counted after 4 days of storage in a growth chamber at 24°C with a 16 h/8 h light/dark photoperiod.

### GFP imaging

GFP fluorescence in the inoculated leaves was monitored by illumination with a handheld UV source (DESAGA). At higher magnifications, GFP fluorescence was detected using a dissecting microscope (Opton IIIRS) equipped with an epifluorescence module. Unless otherwise indicated, the lower epidermal cells of injected leaves were observed at 24 or 72 h after agroinfiltration.

### GFP fluorescence measurement

50 mg of leaf tissue from infiltrated areas were ground in the 1.5 ml tubes in 200 µl of GFP-extraction buffer (150 mM NaCl, 10 mM Tris-HCl, pH 8.0). Then the samples were centrifuged 16 000× g 10 min and 1 ml of GFP-extraction buffer was added to the supernatant. The fluorescence was measured using Quantech fluorometer (ThermoScientific, USA).

### Northern blot analysis

Plant material was ground to a fine powder in liquid nitrogen using a mortar and pestle. Total RNA was extracted from leaves using TRIzol reagent (Invitrogen). Approximately 5 µg of total nucleic acid isolated from mock-treated or virus infected leaves was denatured, separated in 1.5% agarose gels containing 10% formaldehyde in MOPS buffer, pH 7.0, and transferred to a nylon membrane (Hybond-N^+^, Amersham). Membranes were incubated in a pre-hybridization solution containing 6× SSC, 0.5% SDS, 5× Denhardt's reagent and 200 µg/ml tRNA for 4 h at 68°C and probed with a denatured DNA fragment containing the PME coding sequence. Probes were labeled with [α^32^P]-dATP (3000 Ci/mmole) in a PCR reaction.

### Plant infection with TMV

*N. benthamiana* plants withdrawn from the desiccator after exposure to methanol were mechanically inoculated with TMV virions (100 µg/ml) in 50 mM sodium phosphate buffer, pH 7.0, in the presence of Celite, as described previously [106].

### Construction of SSH cDNA libraries

#### RNA isolation and cDNA preparation

Total RNA was isolated from the leaves of control and methanol-exposed *N. benthamiana* [51]. mRNA was purified from total RNA isolated using the PolyATtract mRNA Isolation System I (Promega, USA) according to the protocol supplied along with the kit. Amplified double-stranded cDNA was prepared from methanol- and control-plant RNA using the SMART approach, as described previously [107]. SMART Oligo II oligonucleotide and CDS primers (Table S3) were used for first-strand cDNA synthesis. In both cases, first-strand cDNA synthesis was conducted using 0.3 µg RNA in a total reaction volume of 10 µl. One microliter of 5× diluted first-strand cDNA was then used for PCR amplification with SMART PCR primers. Eighteen PCR cycles (95°C for 7 s, 65°C for 20 s, and 72°C for 3 min), were performed. The SMART-amplified cDNA samples were then further digested by the *Rsa*I endonuclease.

#### Subtraction procedure

Subtractive hybridization was performed using the SSH method in both directions (methanol vs. control and control vs. methanol), as described [108], [109]. Briefly, for each direction, two tester populations were created by the ligation of different suppression adapters (Adapters 1 and 2R). These tester populations were mixed with a 30× excess of driver (driver cDNA had no adaptors) in two separate tubes, denatured, and allowed to re-nature. After the first hybridization, these two samples were mixed and hybridized together. The subtracted cDNA was then amplified by primary and secondary PCR. For the primary PCR, 25 PCR cycles with PCR primer 1 were performed for subtracted methanol cDNA and 25 cycles for subtracted control cDNA. For the secondary (nested) PCR, 10 PCR cycles with nested primers 1 and 2R were performed for both subtracted cDNA samples. The previously described Mirror Orientation Selection (MOS) method [110] was exploited to eliminate type II background from both SSH-generated libraries. In the case of MOS PCR, 22 PCR cycles with MOS PCR primer were performed for subtracted methanol cDNA and 22 cycles were performed for subtracted control cDNA.

#### Construction of subtracted library

Two subtracted cDNA samples enriched with differentially expressed sequences (methanol-specific and control-specific), obtained by MOS PCR, were used to construct the library. In each case, approximately 40 ng of purified cDNA was cloned into the pAtlas vector (pUC base vector) and transformed into *E. coli*. For both libraries, the white to blue colony ratio was 65∶35.

#### Differential screening of subtracted libraries

Ninety-six (one 96-well plate) randomly picked white clones from the tester methanol-specific library and ninety-six (one 96-well plate) randomly picked white clones from the driver control-specific library were used for differential screening. All clones were grown in 100 µl of LB-Amp (75 µg/ml) media for 6 h at 37°C. One-microliter aliquots of each culture were used for PCR amplification with F1S and R1S primers. The plates were subsequently diluted with 20% glycerol and stored at −70°C. Two microliters of each of the PCR-amplified inserts (approximately 100 µg DNA) was arrayed in 96-well format onto duplicated nylon membranes and hybridized with ^32^P-labeled subtracted methanol- and control-subtracted cDNA probes.

#### Virtual Northern blot analysis

Virtual Northern blot analysis was performed to confirm differential screening results. SMART-amplified “driver” (methanol) and “tester” (control) unsubtracted cDNAs were resolved on agarose gels and transferred to Hybond-N membranes. Membranes were hybridized with ^32^P-labeled probes prepared from randomly selected differential clones identified by differential screening. The clones from the methanol-subtracted library and control-subtracted library were used for Virtual Northern blotting. Selected plasmids from 320 clones were purified and sequenced using F1S or R1S plasmid primers.

### Q-PCR analysis of transcript concentrations

Concentrations were determined using a Nanodrop ND-1000 spectrophotometer (Isogen Life Sciences). All RNA samples had a 260∶280 absorbance ratio between 1.9 and 2.1.

After DNAse treatment (Fermentas), 2 µg of denatured total RNA was annealed with 0.1 µg of random hexamers and 0.1 µg of Oligo-dT and incubated with 200 units of Superscript II reverse-transcriptase (Invitrogen, USA) for 50 min at 43°C to generate cDNA. Real-time qPCR was carried out using the iCycler iQ real-time PCR detection system (Bio-Rad, Hercules, CA, USA). Target genes were detected using Eva Green master mix (Syntol, Russia) according to the manufacturer's instructions. The thermal profile for EVA Green real-time qPCR included an initial heat-denaturing step at 95°C for 3 min and 45 cycles with a denaturation step at 95°C for 15 s, an annealing step (amplicon-specific temperatures provided in Table S4) for 30 s and an elongation step at 72°C for 30 s coupled with fluorescence measurement. Following amplification, the melting curves of the PCR products were monitored from 55 to 95°C to determine the specificity of amplification. Each sample was run in triplicate, and a non-template control was added to each run. Target gene mRNA levels were calculated according to the equation proposed by Pfaffl [111]: EtargetΔCt target (sample-reference). PCR efficiency (E) was calculated according to the equation E = 10(−1/slope) by performing the standard curves. Target gene mRNA levels were normalized to the corresponding reference genes (18S and ef-2ά for *N. tabacum*).

### Statistics

Student's *t*-tests were performed using Excel (Microsoft, Redmond, WA). ANOVA tests were performed using SPSS v.18 (IBM Corporation, Somers, NY). *P*-values<0.05 were considered significant.

## Supporting Information

Figure S1 Measurement of the methanol content in the headspace of wounded leaves in the water-drop set-up. *N. benthamiana* leaves (0.5–0.7 g) were rubbed with Celite and loaded into the jar. After 30-, 60- and 180-min incubations, the leaves were removed, and the methanol content in the water drop was measured.
(TIF)

Figure S2 Methanol contents in the sap of leaf tissues from transgenic tobacco. Error bars indicate the SE of data from six independent samples. The *P*-value of unpaired two-tailed Student's *t*-test for statistical significance of the difference between *PME*-transgenic (PME) and wild-type (WT) plants is indicated.
(TIF)

Figure S3 The amino acid sequence of MIG-21. Stretches of repeated amino acids are underlined.
(DOC)

Figure S4 Validation of the expression of the selected MIGs. *N. benthamiana* plants were placed in a hermetically sealed 20-l desiccator with gaseous methanol (160 mg) evaporating from a piece of methanol-soaked filter paper. RNA isolated from leaves of plants stored for 6, 12 or 24 h in this gaseous methanol atmosphere were analyzed by qRT-PCR. Relative quantities of mRNA were normalized to those in control plants stored in a desiccator with water-soaked filter paper. The data shown represent the six independent experiments. The standard error bars are indicated.
(TIF)

Figure S5 The expression of the LOX, PR-3, PR-4, FPS and PAL genes in VOC-treated *N. benthamiana* plants, examined by qPCR. The data shown represent five independent experiments. The standard error bars are indicated.
(TIF)

Figure S6 Necroses in leaves of the *PME*-transgenic tobacco line pro1 (PME) and wild type (WT) tobacco plants at 4 days after injection with *R. solanacearum* (10^8^ cfu/ml). The arrowhead shows the site of injection.
(TIF)

Table S1 Methanol-responsive ESTs from *N. benthamiana*.
(DOC)

Table S2 Bacterial growth in tobacco leaves 4 days after injection with *R. solanacearum* (10^6^ cfu/ml).
(DOC)

Table S3 Oligonucleotides used for SSH.
(DOC)

Table S4 Oligonucleotides used for qPCR.
(DOC)
