# Supplementary figures and images for: Airborne Signals from a Wounded Leaf Facilitate Viral Spreading and Induce Antibacterial Resistance in Neighboring Plants
Source: PLoS Pathog. 2012 Apr 5;8(4):e1002640. doi: 10.1371/journal.ppat.1002640 (PMC3320592; doi:10.1371/journal.ppat.1002640)

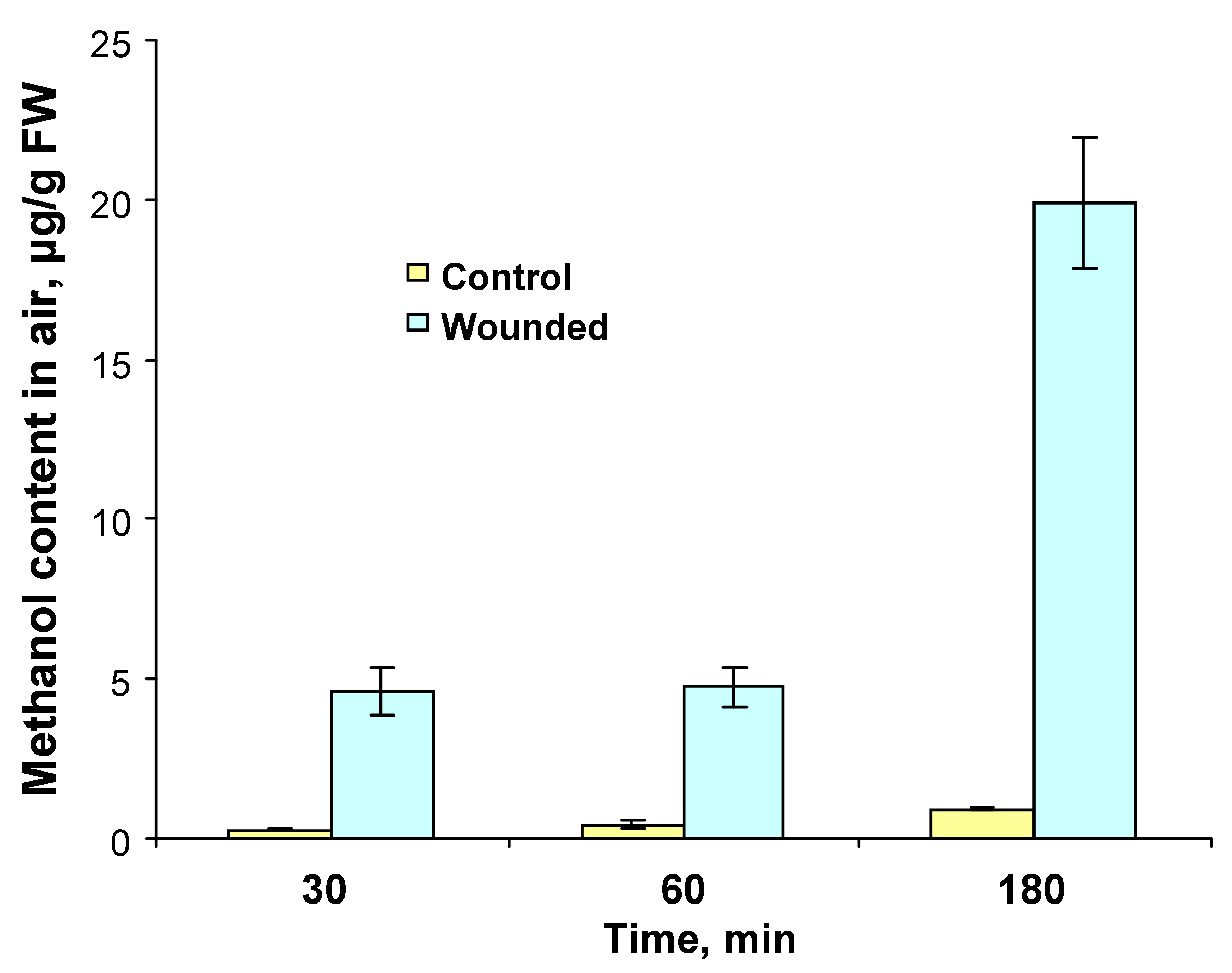

Supplement: Figure S1 — Measurement of the methanol content in the headspace of wounded leaves in the water-drop set-up. N. benthamiana leaves (0.5–0.7 g) were rubbed with Celite and loaded into the jar. After 30-, 60- and 180-min incubations, the leaves were removed, and the methanol content in the water drop was measured. (TIF) [file ppat.1002640.s001.tif]

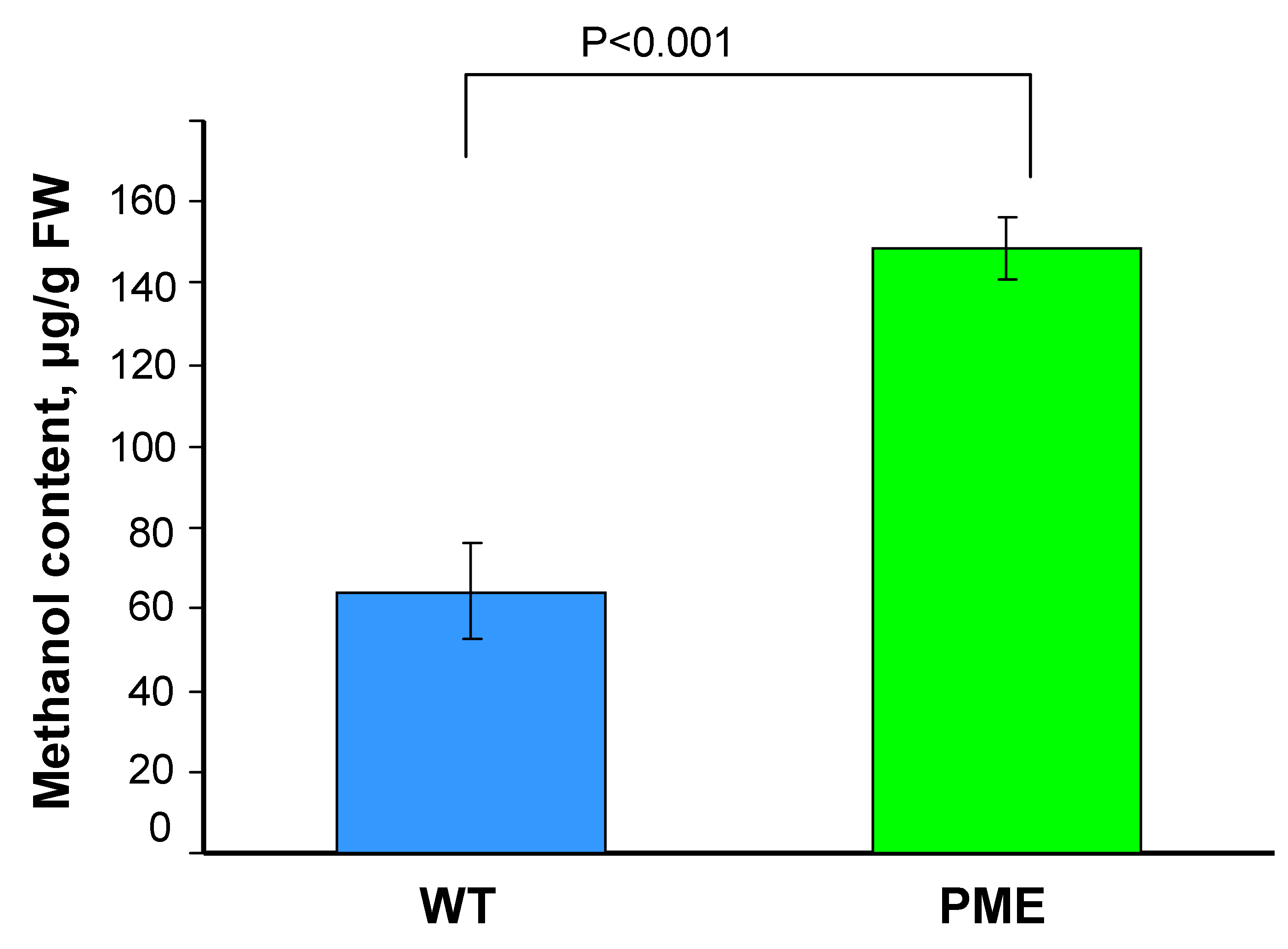

Supplement: Figure S2 — Methanol contents in the sap of leaf tissues from transgenic tobacco. Error bars indicate the SE of data from six independent samples. The P-value of unpaired two-tailed Student's t-test for statistical significance of the difference between PME-transgenic (PME) and wild-type (WT) plants is indicated. (TIF) [file ppat.1002640.s002.tif]

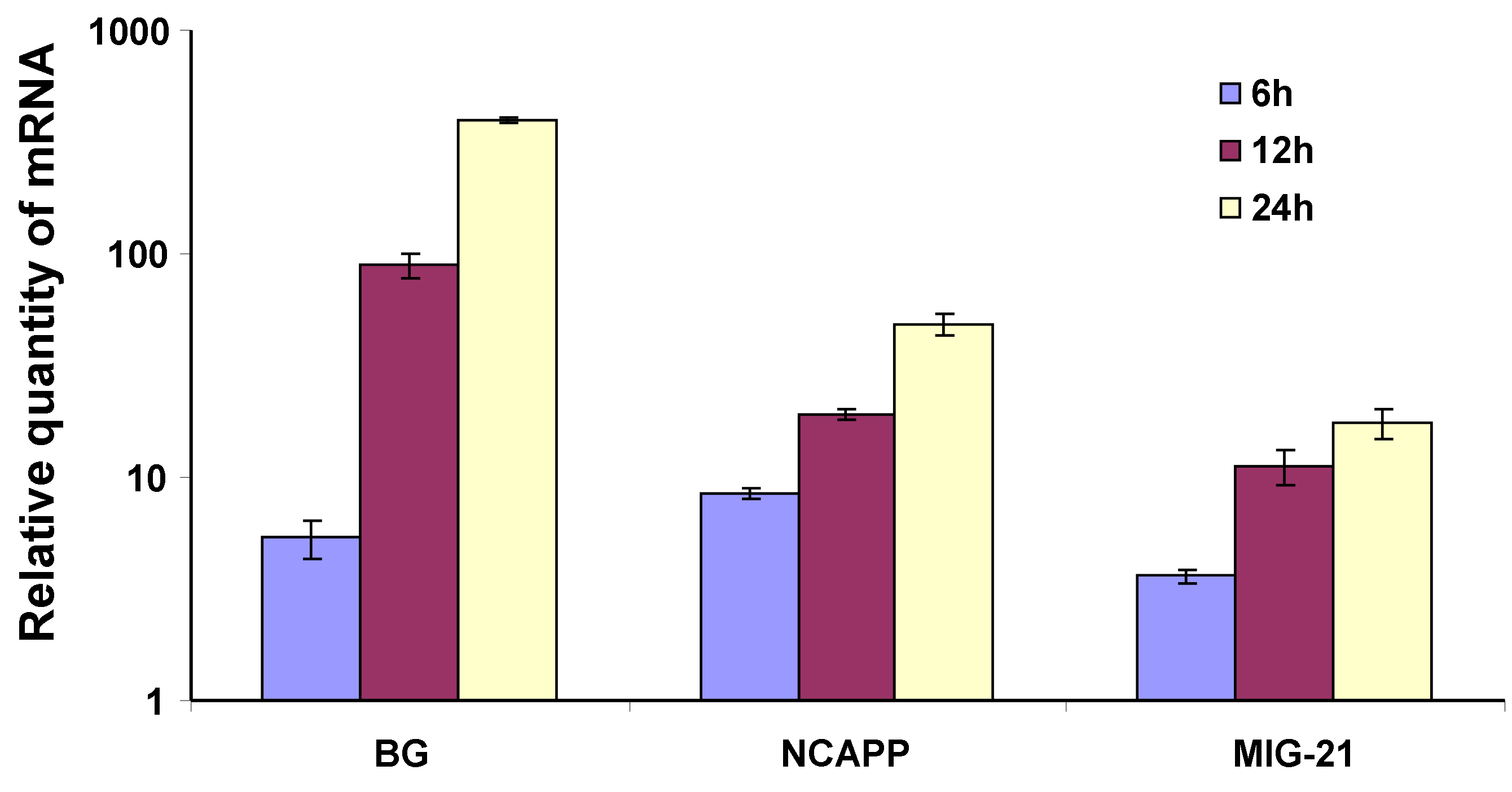

Supplement: Figure S4 — Validation of the expression of the selected MIGs. N. benthamiana plants were placed in a hermetically sealed 20-l desiccator with gaseous methanol (160 mg) evaporating from a piece of methanol-soaked filter paper. RNA isolated from leaves of plants stored for 6, 12 or 24 h in this gaseous methanol atmosphere were analyzed by qRT-PCR. Relative quantities of mRNA were normalized to those in control plants stored in a desiccator with water-soaked filter paper. The data shown represent the six independent experiments. The standard error bars are indicated. (TIF) [file ppat.1002640.s004.tif]

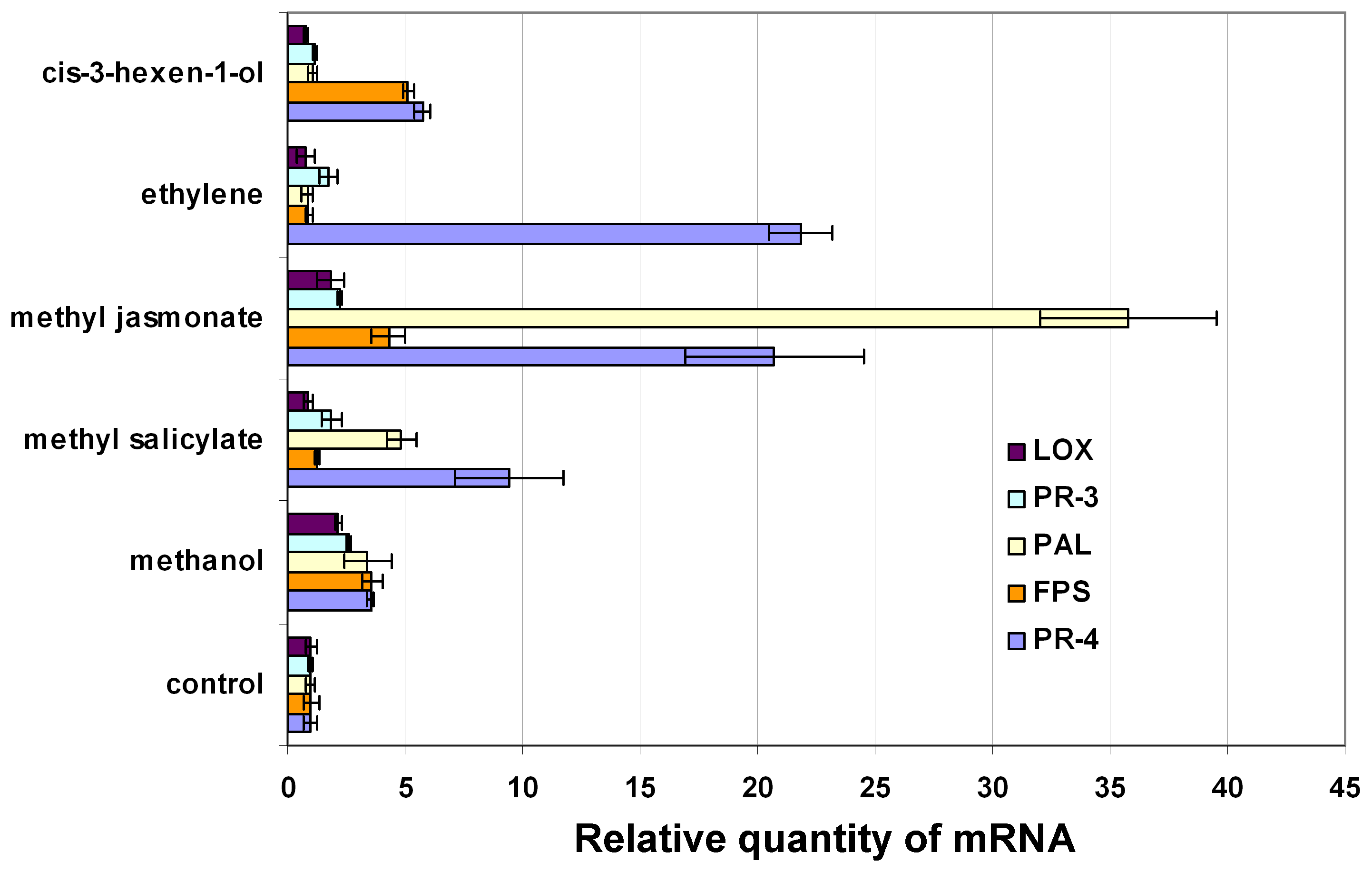

Supplement: Figure S5 — The expression of the LOX, PR-3, PR-4, FPS and PAL genes in VOC-treated N. benthamiana plants, examined by qPCR. The data shown represent five independent experiments. The standard error bars are indicated. (TIF) [file ppat.1002640.s005.tif]

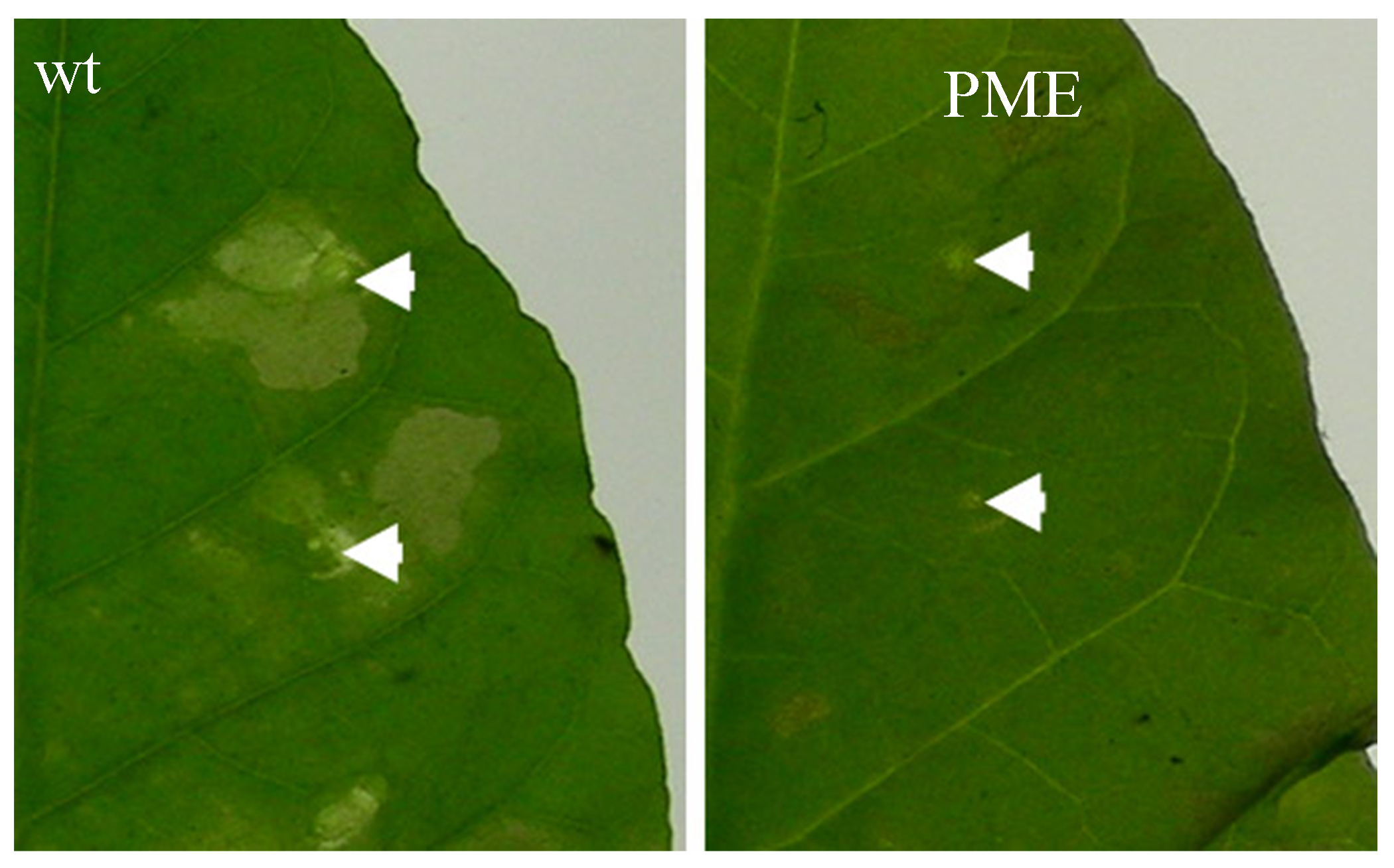

Supplement: Figure S6 — Necroses in leaves of the PME-transgenic tobacco line pro1 (PME) and wild type (WT) tobacco plants at 4 days after injection with R. solanacearum (108 cfu/ml). The arrowhead shows the site of injection. (TIF) [file ppat.1002640.s006.tif]
