# Supplementary material for: Airborne Signals from a Wounded Leaf Facilitate Viral Spreading and Induce Antibacterial Resistance in Neighboring Plants
Source: PLoS Pathog. 2012 Apr 5;8(4):e1002640. doi: 10.1371/journal.ppat.1002640 (PMC3320592; doi:10.1371/journal.ppat.1002640)
Supplement: Figure S3 — The amino acid sequence of MIG-21. Stretches of repeated amino acids are underlined. (DOC) [file ppat.1002640.s003.doc]

MASLQCHKPAQHAPSTLCQKTTTVTCNKANNEHHSFADKMKDMTDKMYHH-50

DSHNHQSACHGTKTQQ*T*AACHGTKTQQSAACHGTKTQQSAASHRTKTQQT-100

ACHGTSANGTKTQLSVACHGTKTQQSAASHGTKTQQTACHGTSATATHAR-150

ACGKKKEGSFMHKMRDQMRSRRNRNKDGSCSDGSDSSSSSSSDESDNENC-200

GRTKNRGSC -209
