# Supplementary material for: Airborne Signals from a Wounded Leaf Facilitate Viral Spreading and Induce Antibacterial Resistance in Neighboring Plants
Source: PLoS Pathog. 2012 Apr 5;8(4):e1002640. doi: 10.1371/journal.ppat.1002640 (PMC3320592; doi:10.1371/journal.ppat.1002640)
Supplement: Table S1 — Methanol-responsive ESTs from N. benthamiana. (DOC) [file ppat.1002640.s007.doc]

| Class target | Functional annotation | Matching with | EST clones (n) | E-value | GenBank accession number |
| --- | --- | --- | --- | --- | --- |
| Upregulated | | | | | |
| Stress, defense, shock-related proteins | Beta-1,3-glucanase, basic PR-2*  Beta-1,3-glucanase (vacuolar isoform) | *N. tabacum*  *N. plumbaginifolia* | 36  15 | 6e-30  2e-66 | FN432035 FN432037 |
|  | Proteinase inhibitor II* | *N. tabacum* | 24 | 1e-42 | FN432036 |
|  | Methanol-induced gene-21 (MIG-21)* | *S. tuberosum* | 22 | 1e-16 | FN432041 |
|  | PME inhibitor* | *N. tabacum* | 7 | 6e-28 | FN432040 |
|  | Elicitor inducible protein | *N. tabacum* | 7 | 5e-23 | FN432735 |
|  | **^§^** 1-aminocyclopropane-1-carboxylic acid oxidase (ACC) oxidase (ACO)* | *S. lycopersicum* | 6 | 7e-21 | FN432033 |
|  | Beta-cyanoalanine synthase | *S. tuberosum* | 5 | 5e-37 | FN432034 |
|  | Metallophosphatase/  diphosphonucleotide phosphatase 1 | *Olea europaea* | 5 | 5e-34 | FN561907 |
|  | BURP domain-containing protein | *S. tuberosum* | 4 | 2e-14 | FN561900 |
|  | NCAPP, non-cell autonomous pathway protein * | *N. tabacum* | 3 | 4e-79 | FN432039 |
|  | Salicylic acid binding catalase | *N. tabacum* | 3 | 2e-56 | FN432738 |
|  | Heat shock protein 70 | *N. tabacum* | 3 | 1e-19 | FN432734 |
|  | Micro-Tom EIN3-binding F-box protein | *S. lycopersicum* | 2 | 0 | FN561909 |
|  | Pathogen- and wound-inducible antifungal protein CBP20 | *N. tabacum* | 2 | 3e-59 | FN432038 |
|  | DnaJ protein | *S. tuberosum* | 2 | 0 | FN561911 |
|  | Peroxidase | *Populus trichocarpa* | 2 | 5e-25 | FN432751 |
|  | Putative stress-induced protein | *S. commersonii* | 1 | 3e-60 | FN564151 |
|  | Dehydration-induced protein | *L. esculentum* | 1 | 4e-25 | FN432731 |
|  | DNA repair protein recA | *A. thaliana* | 1 | 9e-09 | FN432750 |
|  | Serine-type endopeptidase | *A. thaliana* | 1 | 3e-08 | FN561906 |
|  | Yth domain-containing protein | *R. communis* | 1 | 2e-20 | FN561902 |
|  | Xyloglucan endotransglucosylase-hydrolase | *L. esculentum* | 1 | 8e-38 | FN561908 |
|  | Cinnamyl alcohol dehydrogenase 1 | *N. tabacum* | 1 | 0 | FN561912 |
|  | Auxin binding / ubiquitin-protein ligase | *A. thaliana* | 1 | 7e-46 | FN561916 |
|  | Xaa-Pro aminopeptidase 2 | *S. lycopersicum* | 1 | 2e-31 | FN561918 |
|  | DNA double-strand break repair rad50 ATPase | *R. communis* | 1 | 4e-41 | FN564158 |
|  | Nt-RSH2, homolog of bacterial ppGpp synthetase | *N. tabacum* | 1 | 0 | FN564157 |
| Energy flow pathway proteins | Phosphoenolpyruvate carboxykinase | *L. esculentum* | 8 | 2e-20 | FN432757 |
|  | Plastidic aldolase | *N. paniculata* | 6 | 3e-44 | FN432768 |
|  | Calcineurin-like phosphoesterase | *A. thaliana* | 4 | 3e-31 | FN432740 |
|  | NADPH:protochlorophyllide oxidoreductase | *N. tabacum* | 4 | 8e-30 | FN561905 |
|  | Ribulose bisphosphate carboxylase activase | *N. tabacum* | 3 | 0 | FN561924 |
|  | Phosphatase 2C homolog | *Mesembryanthemum crystallinum* | 3 | 9e-32 | FN432754 |
|  | F1-ATPase alpha subunit | *Dulacia candida* | 3 | 2e-26 | FN432732 |
|  | Caleosin | *Sesamum indicum* | 3 | 2e-25 | FN432729 |
|  | Cationic peroxidase | *Stylosanthes humilis* | 2 | 6e-49 | FN432032 |
|  | Glycolate oxidase | *N. tabacum* | 2 | 1e-38 | FN432743 |
|  | Putative metallophosphatase | *Lupinus luteus* | 2 | 3e-58 | FN432746 |
|  | mitochondrial ATP-dependent Clp protease, proteolytic subunit | *R. communis* | 2 | 3e-16 | FN432733 |
|  | Nucleotide Pyrophosphatase  /phosphodiesterase | *Z. mays* | 2 | 6e-13 | FN433836 |
|  | RUBIsco binding protein | *L. esculentum* | 1 | 0 | FN564152 |
|  | Oxygen-evolving enhancer protein 2-2, chloroplastic | *N. tabacum* | 1 | 2e-58 | FN564153 |
|  | Chloroplast oxygen-evolving protein | *N. benthamiana* | 1 | 0 | AY887536.1 |
|  | Beta-galactosidase | *L. esculentum* | 1 | 1e-28 | FN432744 |
|  | Chlorophyll a/b-binding protein | *N. tabacum* | 1 | 2e-47 | FN432759 |
|  | Ribulose bisphosphate carboxylase | *N. plumbaginifolia* | 1 | 4e-15 | FN432730 |
|  | Photosystem II CP47 chlorophyll apoprotein | *Geranium palmatum* | 1 | 6e-55 | FN564155 |
|  | Metal ion binding protein | *R. communis* | 1 | 3e-20 | FN564154 |
|  | Putative chloroplast cysteine synthase 1 precursor | *N. tabacum* | 1 | 0 | FN561901 |
|  | Chloroplast-specific ribosomal protein | *L. esculentum* | 1 | 2e-80 | FN564156 |
|  | RNA recognition motif (RRM)-containing protein | *A. thaliana* | 1 | 4e-12 | FN561898 |
|  | Phosphatidylinositol 3- and 4-kinase family protein | *A. thaliana* | 1 | 5e-53 | FN561917 |
|  | Catalytic/ isocitrate lyase | *Coprinopsis cinerea* | 1 | 1e-48 | FN561921 |
| Anabolism | Ribosomal protein L23 | *N. tabacum* | 3 | 3e-38 | FN433837 |
|  | Poly(A)-binding protein | *N. tabacum* | 1 | 2e-78 | FN561915 |
|  | Spermidine synthase | *N. tabacum* | 1 | 0 | FN561910 |
|  | Sterol carrier protein 2 | *O. sativa* | 1 | 4e-21 | FN561899 |
|  | P40-like protein | *S. tuberosum* | 1 | 3e-62 | FN561904 |
|  | S-adenosylmethionine decarboxylase | *Prunus persica* | 2 | 3e-17 | FN432745 |
| Ion transporters | Ca2+/H+-exchanging like protein | *A. thaliana* | 3 | 2e-07 | FN433835 |
|  | Ca2+ antiporter/cation exchanger | *Populus trichocarpa* | 2 | 2e-06 | FN433834 |
| Downregulated | | | | | |
| Stress, defense proteins | Lipid transfer protein (LTP1, 3, 4) | *N. tabacum* | 8 | 3e-58 | D13952.1 |
|  | Glycine-rich protein precursor | *N. tabacum* | 4 | 2e-29 | M37152.1 |
|  | Antimicrobal peptide Snakin2 | *S. tuberosum* | 2 | 4e-33 | AJ312424 |
| Homeostasis, metal tolerance | Metallothionein-like protein type 2  Metallothionein | *N. tabacum*  *N. glutinosa* | 1  1 | 2e-13  6e-34 | AJ299253.1  U46543.1 |

*Methanol-induced ESTs selected for further analysis

^§^ Arabidopsis methanol-induced ESTs identified by Downie *et al.* (62) are highlighted in yellow.
