# Supplementary material for: Airborne Signals from a Wounded Leaf Facilitate Viral Spreading and Induce Antibacterial Resistance in Neighboring Plants
Source: PLoS Pathog. 2012 Apr 5;8(4):e1002640. doi: 10.1371/journal.ppat.1002640 (PMC3320592; doi:10.1371/journal.ppat.1002640)
Supplement: Table S2 — Bacterial growth in tobacco leaves 4 days after injection with R. solanacearum (106 cfu/ml). (DOC) [file ppat.1002640.s008.doc]

| Tobacco  plant | *R. solanacearum* number* (cfu/cm^2^)x10^6^ | | | |
| --- | --- | --- | --- | --- |
|  | 0h | 72h | Ratio  72h/0h | Relative  growth, % |
| Wild type | 1.09±0.02 | 3.07±0.02 | 2.82±0.01 | 182 |
| PME transgenic | 1.05±0.01 | 1.19±0.07 | 1.13±0.02 | 13 |

*** The data were obtained from six independent samples and in three independent experiments
