# Supplementary material for: Airborne Signals from a Wounded Leaf Facilitate Viral Spreading and Induce Antibacterial Resistance in Neighboring Plants
Source: PLoS Pathog. 2012 Apr 5;8(4):e1002640. doi: 10.1371/journal.ppat.1002640 (PMC3320592; doi:10.1371/journal.ppat.1002640)
Supplement: Table S3 — Oligonucleotides used for SSH. (DOC) [file ppat.1002640.s009.doc]

| SMART Oligo II A oligonucleotide | 5’-AAGCAGTGGTATCAACGCAGAGTACGCrGrGrG-3’ |
| --- | --- |
| SMART CDS primer II A | 5’-AAGCAGTGGTATCAACGCAGAGTA-d(T)30-3’ |
| SMART PCR primer II A | 5’-AAGCAGTGGTATCAACGCAGAGT-3’ |
| Adapter 1 | 5’-CTAATACGACTCACTATAGGGCTCGAGCGGCCGCCCGGGCAGGT-3’  3’-GGCCCGTCCA-5’ |
| PCR primer 1 | 5'-CTAATACGACTCACTATAGGGC-3' |
| Nested primer 1 | 5'-TCGAGCGGCCGCCCGGGCAGGT-3' |
| Adapter 2R | 5'-CTAATACGACTCACTATAGGGCAGCGTGGTCGCGGCCGAGGT-3'  3'-GCCGGCTCCA-5' |
| Nested primer 2R | 5'-AGCGTGGTCGCGGCCGAGGT-3' |
| F1S plasmid primer | 5'-AGTACGCTCAAGACGACAGAA-3' |
| R1S plasmid primer | 5'-AAAGCAGTGGTAACAACGCAG-3' |
| MOS PCR primer | 5'–GGTCGCGGCCGAGGT-3' |
