# Supplementary material for: Airborne Signals from a Wounded Leaf Facilitate Viral Spreading and Induce Antibacterial Resistance in Neighboring Plants
Source: PLoS Pathog. 2012 Apr 5;8(4):e1002640. doi: 10.1371/journal.ppat.1002640 (PMC3320592; doi:10.1371/journal.ppat.1002640)
Supplement: Table S4 — Oligonucleotides used for qPCR. (DOC) [file ppat.1002640.s010.doc]

| **Gene** | **Forward Primer** | **Reverse Primer** | **PCR Size, Bp** | **Acquisition temperature,^o^C** |
| --- | --- | --- | --- | --- |
| EF1alfa | GTCGTGTCCTGATTGTTG | GGTAGCATCCATCTTGTTG | 155 | 56 |
| 18S rRNA | ACGGCTACCACATCCAAG | ACTCATTCCAATTACCAGACTC | 116 | 50 |
| PR-3 | GCCAAGATGTAGGAGAAGG | GGCAACCACCAGAAGATG | 150 | 50 |
| PAL | GCACCATCACCGCCTCTG | TAACTCCAGCAACACGAAACG | 138 | 52 |
| FPS | GATGGCATACTTCTCCGCAACC | AGAGGCAGTCTGGAACTCAACC | 117 | 52 |
| PR-4 | ACAGGATAATAAGCCATCAAG | CATTCAACATACCACAATACC | 200 | 53 |
| LOX | GAGAGCAGATAGAGGATAAGTTGGATG | GAGCAGAGTTCTTGAGGCATAGG | 158 | 54 |
| PI-II | ATGTACCTCGCCAAACTTAC | CTCCATCTATTTATAGCAAACG | 160 | 54 |
| NCAPP | CTATGTCTTCAAAGATTAGTCTG | GGACGGAAATGATTGTGGC | 159 | 50 |
| PMEi | CAGATTAACAAAAAGCTTAGGG | ACTCCGTATTTGTATCTCCG | 160 | 54 |
| PME | ATCCTTGGATTCCGGCAAGAACGT | AAACACTTGCAATTGTAGAGTAAC | 180 | 54 |
| PR-2 | GATTGTTGTGTCCGAGAGTG | CCAGTTCAGGGTTCTTGTTG | 188 | 52 |
| MIG-21 | AGGAAGGCAGTTTCATGCATAA | GCCTCTTATTTGTTTGAGTTATGCCTC | 199 | 56 |
